# Acupuncture for menstruation-related migraine prophylaxis: A multicenter randomized controlled trial

**DOI:** 10.3389/fnins.2022.992577

**Published:** 2022-08-26

**Authors:** Lu Liu, Claire-Shuiqing Zhang, Hui-Lin Liu, Fan He, Tian-Li Lyu, Lin Zeng, Luo-Peng Zhao, Mi-Na Wang, Zheng-Yang Qu, Li-Min Nie, Jia Guo, Xiao-Zhe Zhang, Yong-Hui Lu, Ke-Lun Wang, Bin Li, Xiang-Hong Jing, Lin-Peng Wang

**Affiliations:** ^1^Department of Acupuncture and Moxibustion, Beijing Key Laboratory of Acupuncture Neuromodulation, Beijing Hospital of Traditional Chinese Medicine, Capital Medical University, Beijing, China; ^2^School of Health and Biomedical Sciences, RMIT University, Melbourne, VIC, Australia; ^3^School of Information Management, Wuhan University, Wuhan, China; ^4^Research Centre of Clinical Epidemiology, Peking University Third Hospital, Beijing, China; ^5^Beijing Hospital of Traditional Chinese Medicine, Beijing Institute of Traditional Chinese Medicine, Capital Medical University, Beijing, China; ^6^Institute of Acupuncture and Moxibustion, China Academy of Chinese Medical Sciences, Beijing, China; ^7^Traditional Chinese Medicine Department, Peking University Third Hospital, Beijing, China; ^8^Department of Pain Management, Beijing Tiantan Hospital, Capital Medical University, Beijing, China; ^9^Department of Acupuncture and Moxibustion, Xiyuan Hospital, China Academy of Chinese Medical Sciences, Beijing, China; ^10^Department of Health Science and Technology, Centre for Sensory-Motor Interaction, Aalborg University, Aalborg, Denmark

**Keywords:** acupuncture, alternative medicine, efficacy, menstruation-related migraine, prophylaxis, safety

## Abstract

**Objective:**

The aim of this study was to evaluate the efficacy of acupuncture, an alternative medicine therapy, as a preventive treatment for menstruation-related migraine (MRM).

**Patients and methods:**

This was a prospective, multicenter, double-dummy, participant-blinded, randomized controlled clinical trial conducted in China between 1 April 2013, and 30 April 2014. The participants were enrolled from four study centers and randomized to into either the acupuncture group, which received 24 sessions of acupuncture at traditional acupoints plus placebo, or the medication group, which received sham acupuncture plus naproxen. The primary endpoint was change from the baseline average number of migraine days per perimenstrual period over cycles 1−3. The secondary endpoints included changes from the baseline average number of migraine days outside the perimenstrual period, mean number of migraine hours during and outside the perimenstrual period, mean visual analog scale score during and outside the perimenstrual period, ≥50% migraine responder rate, and the proportion of participants who used acute pain medication over cycles 1−3 and 4−6.

**Results:**

A total of 172 women with MRM were enrolled; 170 in the intention-to-treat analyses. Our primary outcome reported a significant between-group difference that favored the acupuncture group (95% CI, 0.17–0.50; *P* < 0.001), with the average reduction of migraine days per perimenstrual period from the baseline was 0.94 (95% CI, 0.82–1.07) in the acupuncture group and 0.61 (95% CI, 0.50–0.71) in the medication group over cycles 1−3.

**Conclusion:**

This study showed that compared to medication, acupuncture reduces the number of migraine days experienced by patients with MRM. For patients who received the acupuncture treatment over three cycles, the preventive effect of the therapy was sustained for six cycles.

**Clinical trial registration:**

[https://www.isrctn.com/ISRCTN57133712], identifier [ISRCTN15663606].

## Introduction

Migraine affects 18.9% of women globally (Collaborators GBDH, 2018), and its occurrence in women is frequently associated with menstruation. Menstrual migraine affects 20% of female migraineurs and most of the attacks are migraines without aura (Vetvik et al., 2014). Menstrual migraine can be divided into two subtypes: pure menstrual migraine and menstruation-related migraine (MRM), with most of the cases being MRM. Approximately 60% of female migraineurs report an association between migraine and menstruation (Pavlović et al., 2015). According to the International Classification of Headache Disorders (ICHD) (versions II, III beta, and III), MRM is defined as attacks of migraine without aura, occurring between two days before the onset of menstruation and the third day of menstruation (five-day window) in at least two out of three menstrual cycles, with additional attacks of migraine at other times of the cycles (Headache Classification Subcommittee of the International Headache Socitey [IHS], 2004; Headache Classification Committee of the International Headache Society [IHS], 2013; Headache Classification Committee of the International Headache Society [IHS], 2018). MRM causes a significant public health burden, particularly during women’s reproductive years (Pavlović et al., 2015).

The clinical management of MRM is challenging. The migraine attacks that occur during the perimenstrual period tend to be more painful, longer lasting, more disabling, and accompanied by more severe nausea than those that occur outside the perimenstrual period (Vetvik et al., 2015). In addition, these attacks are less responsive to medication than non-menstrual migraines (Granella et al., 2004; MacGregor et al., 2010; Pinkerman and Holroyd, 2010). MRM treatments are classified as acute, short-term prophylaxis, or daily prevention treatment. Pharmacotherapy, including administration of drugs such as aspirin, naproxen, triptans, estrogen, magnesium, and dihydroergotamine, is recommended for the prevention of MRM (Allais et al., 2018). Non-steroidal anti-inflammatory drugs, which are considered the first-line treatment option for migraines, have been widely used for short-term prophylaxis of MRM (Newman and Yugrakh, 2014; Maasumi et al., 2017) owing to their high efficacy and ability to prevent other forms of perimenstrual pain, such as dysmenorrhea (Pringsheim et al., 2008; Allais et al., 2012; Newman and Yugrakh, 2014; Maasumi et al., 2017). However, the use of non-steroidal anti-inflammatory drugs or other preventive treatments are often associated with an increased risk of adverse events (AEs), including cardiovascular disorders, gastrointestinal bleeding, menstrual irregularity, and diarrhea (MacGregor, 2015). In addition, excessive use of analgesics or specific anti-migraine treatments may cause medication-overuse headaches and an increase in the frequency of headaches (Bigal and Lipton, 2008; Bigal et al., 2009). Owing to these limitations associated with conventional treatments, efforts have been made to identify other effective and low-risk interventions for MRM.

Acupuncture is a commonly researched and widely accepted complementary and alternative medicine therapy used for the treatment of migraine (Linde K. et al., 2005; Diener et al., 2006; Wang et al., 2011; Li et al., 2012; Coeytaux and Befus, 2016; Linde et al., 2016; Zhao et al., 2017; Xu et al., 2020). However, only one former rigorous clinical trials by Linde et al. reported the efficacy of acupuncture treatment for MRM, which reminds the lack of researching in both acupuncturing efficiency and MRM (Linde M. et al., 2005). Therefore, we conducted a prospective, randomized, clinical trial under conditions similar to those of routine care to evaluate the efficacy of acupuncture as a preventive treatment for MRM.

## Materials and methods

### Ethical considerations, protocol approvals, and registrations

The protocol of this clinical trial was registered in International Standard Randomized Controlled Trial Number (ISRCTN) with registry no. ISRCTN57133712, and ethical approval was obtained from the Research Ethical Committee of Beijing Hospital of Traditional Chinese Medicine (ref: 201212) prior to the commencement of the trial. The study was conducted in accordance with the principles of the Declaration of Helsinki, and reported according to the guidelines of the Consolidated Standards of Reporting Trials (Moher et al., 2010), as well as the Standards For Reporting Interventions In Controlled Trials Of Acupuncture (MacPherson et al., 2002) guidelines. The methods are fully reported in the published protocol (Supplementary material 1; Zhang et al., 2013). All the included patients provided written informed consent prior to participation.

### Study design and patients

This was a prospective, multicenter, double-dummy, participant-blinded, randomized controlled clinical trial that consisted of three phases: a baseline phase (cycle -3 to cycle 0), a treatment phase (cycle 1 to cycle 3), and a follow-up phase (cycle 4 to cycle 6). The study was conducted at the departments of acupuncture and pain management of the following centers in China: Beijing Hospital of Traditional Chinese Medicine, Peking University Third Hospital, Beijing Tiantan Hospital, and Xiyuan Hospital. One experienced neurologist in each center assessed the eligibility of all potential participants according to predefined inclusion/exclusion criteria and provided detailed explanation of the trial design to the participants.

The inclusion criteria were as follows: (Collaborators GBDH, 2018) patients diagnosed with MRM according to the diagnostic criteria of the ICHD, version II (Headache Classification Subcommittee of the International Headache Socitey [IHS], 2004; Vetvik et al., 2014) patients with regular menstrual cycles (25−35 days); (Pavlović et al., 2015) patients who can predict the onset of menstruation and perimenstrual migraine attacks within three days prior; (Headache Classification Subcommittee of the International Headache Socitey [IHS], 2004) patients who experience repeated migraine attacks, with the frequency of non-menstrual migraine being more than once a month; and (Headache Classification Committee of the International Headache Society [IHS], 2013) patients who provided written informed consent. The exclusion criteria were as follows: (Collaborators GBDH, 2018) patients with chronic migraine, tension headache, cluster headache, and other primary headaches; (Vetvik et al., 2014) patients with secondary headache and other neurological diseases; (Pavlović et al., 2015) patients with relatively severe systemic diseases (cardiovascular disease, acute infectious disease, hematopathy, endocrinopathy, and allergies); (Headache Classification Subcommittee of the International Headache Socitey [IHS], 2004) patients with headache caused by otorhinolaryngological diseases or intracranial pathological changes; (Headache Classification Committee of the International Headache Society [IHS], 2013) patients taking oral contraceptives and pregnant or lactating patients; (Headache Classification Committee of the International Headache Society [IHS], 2018) patients who used prophylactic migraine medication in the past 3 months; and (Vetvik et al., 2015) patients involved in other clinical trials.

### Study procedures

Eligible participants were randomly assigned into two treatment groups: the acupuncture group, which received acupuncture at traditional acupoints plus placebo naproxen, and the medication group, which received sham acupuncture at non-effective acupoints plus naproxen. Participants were allowed to take acute pain medication during the entire trial and were required to record the details of the medication taken.

#### Randomization and blinding

Eligible participants were randomly assigned at a 1:1 ratio to the acupuncture or medication groups. Central randomization, conducted using an online or messaging system, was performed by the Research Center of Clinical Epidemiology affiliated to Peking University. Randomization was stratified according to centers with a fixed block size of four.

The placebo medication was made to be identical to actual naproxen tablets in terms of taste, smell, and appearance. We used a sham acupuncture method that produced the same stimulation as true acupuncture to ensure that all the participants were blinded to their group allocation. Participants in different groups were treated separately and blinded to the type of acupuncture they received. All outcome assessors and trial statisticians were blinded to the group allocations throughout the duration of the trial. Only the acupuncturists who administered true or sham acupuncture treatment were aware of the participants’ group allocation. The investigators, researchers, and participants were all blinded during the study until the randomization code was broken at the end of the trial or if serious adverse events occurred during the study period. A standard operating procedure and relevant documents were provided to all trial centers to ensure consistency in terms of blinding.

#### Clinical assessments

Participants were required to complete a headache diary from the baseline phase to the end of the follow-up phase. The participants recorded the details of their migraine attacks, including the time the headaches started and ceased, the intensity, frequency, location (the forehead, top, temporal, and back of the head), and cause of the headache, and the concomitant symptoms of each migraine attack. Additionally, participants were required to record information regarding their menstruation and the dates they received acupuncture treatments in their headache diaries. If acute pain medications were taken, participants were required to document the name and dosage of the medicine, the time it was taken, time of pain relief, and the side effects experienced. The headache diary for each cycle was collected by researchers who were blinded to the participants’ group allocation.

The participants were also required to report any AEs they experienced. The causality of the AEs and their association with acupuncture or the trial medication were determined by the trial clinicians.

#### Interventions

The intervention scheme of this trial was determined according to the consensus of experts and the results of a previous pilot study (Li et al., 2011). The acupuncture treatment methods were developed based on the information in classical and modern literature (Yang, 1995; Deng and Huang, 2004) and the results of previous research on the treatment of migraine using acupuncture (Wang et al., 2011). All acupuncture treatments (true or sham) were administered by acupuncturists who are registered with the Ministry of Health of the People’s Republic of China and have more than 20 years of clinical experience. In each trial center, only one acupuncturist administered all the acupuncture treatments to ensure consistency. Prior to the commencement of the trial, all the acupuncturists received training on the purpose and design of the trial, treatment strategies, and quality control.

Naproxen sustained-release tablets (250 mg/tablet) and the placebo medication were provided by the Diao Group Chengdu Pharmaceutical LTD., Chengdu, China. During the three-cycle treatment phase, participants started to take naproxen or placebo (two tablets, once per day) three days before each predicted onset of menstruation and continued until the end of each menstrual cycle. If menstruation began later than predicted, the treatment was not adjusted. If MRM occurred earlier than predicted, participants were asked to begin taking naproxen (or placebo) immediately and one day earlier in the next cycle to provide prophylactic coverage. Considering that the duration of menstruation may vary, the timing of the treatments of the participants was determined based on the information collected from their baseline headache diaries. Variability in the onset of menstruation and migraine during the treatment phase was not taken into account. In addition, participants were allowed to take acute pain medication during the entire trial and required to record the details of the medication.

Ten to twelve sterile disposable steel needles (Hwato Needles, made in Suzhou, China; gauge and size: 0.25 mm × 25 mm for head points, 0.3 mm × 40 mm for limb and abdomen points) were used in each session of the true and sham acupuncture treatments. At each point, the needle was inserted 10 mm to 15 mm into the skin and manipulated using rotation methods to produce a characteristic sensation known as “de qi” (tenseness around the needle felt by the practitioner and numbness, distension, soreness, and heaviness around the point felt by the patient).

##### Treatment of the acupuncture group

In the acupuncture group, participants were administered with true acupuncture plus placebo naproxen. The acupuncture therapy consisted of preventive treatment (two sessions each week) and premenstrual conditioning treatment (at least three sessions during the 10 days before the predicted onset of each menstruation), administered for three cycles. Each session lasted for 30 min. The acupoints (Supplementary Figure 1 in Supplementary material 2) used for preventive treatment included both standard and additional points. The standard points were GV20 (Baihui), GV24 (Shenting), GB13 (Benshen), GB8 (Shuaigu), TE20 (Jiaosun), and GB20 (Fengchi). Additional points were chosen individually depending on the syndrome differentiation of meridians in the headache region. The additional points were TE5 (Waiguan) and GB34 (Yanglingquan) for Shaoyang headache, LI4 (Hegu) and ST44 (Neiting) for Yangming headache, BL60 (Kunlun) and SI3 (Houxi) for Taiyang headache, LR3 (Taichong) and GB40 (Qiuxu) for Jueyin headache, PC6 (Neiguan) for nausea and vomiting, and LR3 (Taichong) for dysphoria and susceptibility to rage. For premenstrual conditioning, each participant received treatment at the standard acupoints KI12 (Dahe), CV3 (Zhongji), and ST29 (Guilai). All the selected acupoints were determined based on the findings of our previous research (Wang et al., 2011).

##### Treatment of the medication group

Participants assigned to the medication group received sham acupuncture treatment plus true naproxen. Sham acupuncture was administered using the same methods used for true acupuncture but on non-effective acupoints. The selection of non-effective acupoints was based on the following rules: (Collaborators GBDH, 2018) acupoints defined as unrelated to headache or menstruation based on the information in a vast amount of Chinese medicine reference books (26 ancient Chinese books of acupuncture, three Chinese acupuncture textbooks, and more than 100 acupuncture research literatures); (Vetvik et al., 2014) 15 acupoints (Supplementary Table 1 in Supplementary material 2) in the vicinity of the elbow and knee joints were selected, whereas the acupoints on the head, hands, feet, and trunk were excluded. To mimic the nature of selecting points based on syndrome differentiation, the 15 sham points in the vicinity of the elbow and knee joints were further randomly assigned into three subgroups, B, C, and D. Each subgroup had two points on the arms and three points on the legs (Supplementary Table 2 in Supplementary material 2). The participants in the medication group were further randomly assigned into one of these three subgroups through a central randomization system.

#### Outcome measures

The primary outcome measure was change from the baseline average number of migraine days per perimenstrual period over cycles 1−3. The secondary outcome measures were changes from the baseline average number of migraine days outside the perimenstrual period, mean number of migraine hours during and outside the perimenstrual period, mean visual analogue scale (VAS) score recorded during and outside the perimenstrual period, ≥50% migraine responder rate, which was defined as the proportion of participants who achieved ≥50% reduction in the number of migraine days, and the proportion of participants who used acute pain medication over cycles 1−3 and 4−6.

Data regarding migraine days, migraine hours, and VAS scores were extracted from the completed headache diaries for each cycle from the start of the baseline phase to the end of the follow-up phase. In the diary, participants documented migraine days, migraine hours, and the intensity and time of each attack. For cycles 1−3 and 4−6, migraine days were calculated as the cycle average, whereas mean migraine hours and mean VAS scores were calculated as the daily average.

#### Safety assessments

At the baseline assessment and the end of the treatment phase, all the participants underwent general medical and neurological examinations carried out by clinicians, in addition to clinical laboratory tests for full blood, liver function, kidney function, and chemistry evaluations. During the trial period, participants were required to document the AEs they experienced in their headache diaries. Medication- or acupuncture-related AEs were documented with full details, and clinicians, including neurologists and acupuncturists, evaluated the severity of each AE. If serious adverse events occurred, the data safety and monitoring committee, which had the right to terminate the trial, adjudicated the severity of the AE reported by the investigators.

#### Credibility of the blinding test

To confirm the blinding of the participants, a blinding test questionnaire was administered to all the participants in the middle of the treatment phase (cycle 1.5) and at the end of the treatment phase (cycle 3). The participants were asked to guess which group they were allocated to.

#### Statistical analysis

Based on the information from a previous pilot study (Li et al., 2011), we estimated that the number of migraine days over 12 weeks would be 3.1 (standard deviation [SD], 2.7) days in the acupuncture group and 5.2 (SD, 4.4) days in the medication group. With a two-sided significance level of 5% and a power of 90%, 68 participants would be required for each group, as calculated using PASS 2008 software (NCSS, Kaysville, UT, United States). Considering an estimated loss-to-follow-up rate of 20%, we planned to enroll a total of 172 participants (86 participants per group) in the study.

The analysis plan was determined before the study was conducted (Supplementary material 1). The baseline characteristics and clinical outcomes described were based on the intention-to-treat (ITT) population, which included participants who received at least one treatment and had at least one primary outcome measure (*n* = 170). We performed sensitivity analyses using the per-protocol (PP) set, which included all randomized participants who had no major protocol deviation. We also performed safety analysis using the safety set, which included all randomized participants who received at least one session of acupuncture.

The primary outcome was analyzed according to the ITT principle. The change from the baseline average number of migraine days per perimenstrual period over cycles 1−3 was analyzed by fitting a mixed-effects model using the baseline value as a covariate, treatment as a fixed effect, and center as a random effect. The same approach was used for the analysis of the secondary outcomes, which included the changes from the baseline average number of migraine days outside each perimenstrual period, mean number of migraine hours during and outside the perimenstrual period, and the mean VAS score recorded during and outside the perimenstrual period. For other continuous variables, comparisons between treatment groups were assessed using the *t*-test or Wilcoxon rank-sum test as appropriate. Categorical variables were compared using the chi-square test or Fisher’s exact test as appropriate. Kappa analysis was used to determine whether participants correctly guessed their group allocation at a higher rate than would be expected by chance.

Missing data on the primary and secondary outcomes were imputed using the last observation carried forward (LOCF) method. To examine the sensitivity of the LOCF method, we performed a sensitivity analysis using multiple imputation methods (Supplementary Appendix 1 and Supplementary Table 3 in Supplementary material 2) under the “missing at random” assumption for missing primary outcome data (Supplementary Appendix 2 in Supplementary material 2).

There are between- and within-woman variations in menstrual cycle lengths that could inherently confound the outcomes. To evaluate this potential confounder, we performed a sensitivity analysis using menstrual cycle length as a control variable in a mixed-effects model (Supplementary Appendix 3 in Supplementary material 2).

An independent statistician who was blinded to the group allocation performed all the statistical analyses using SAS statistical software (SAS Institute, Cary, NC, United States). For both continuous and categorical variables, 95% confidence intervals (CI) were calculated as appropriate. All the statistical comparisons were two sided, and *P* < 0.05 considered significant.

## Results

**FIGURE 1:**
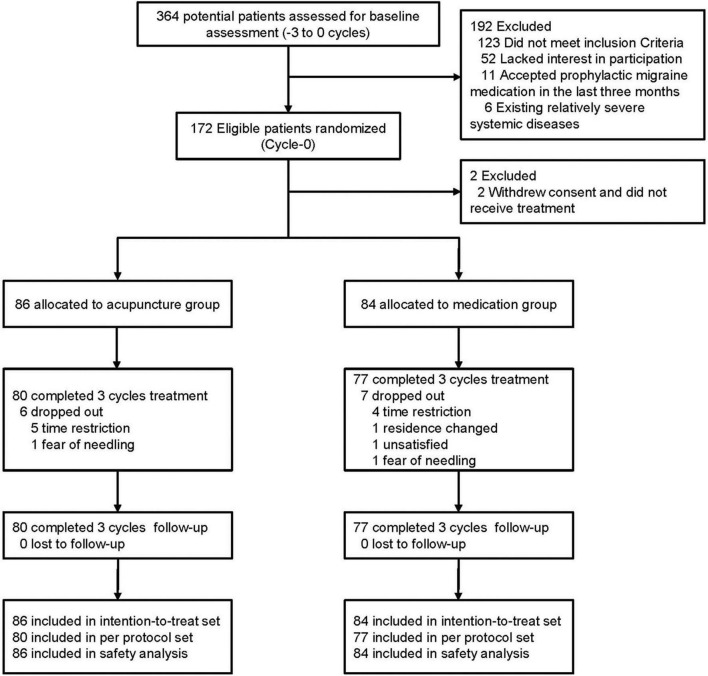
Flowchart of the screening, enrollment, randomization, and follow-up of patients.

Between April 2013 and April 2014, a total of 364 female migraineurs provided informed consent and were screened for eligibility for this study. After exclusion of 192 ineligible patients with detailed reasons recorded and reported (Supplementary Figure 1 and Supplementary Table 7 in Supplementary material 2), a total of 172 participants were randomized into the acupuncture and medication groups. Two participants in the medication group were excluded because they withdrew their consent and did not receive treatment. Thus, 170 participants (86 in the acupuncture group, 84 in the medication group) were included in the ITT population (Figure 1). During the treatment phase, 13 participants dropped out of the study (dropout rate, 7.65%; acupuncture group: *n* = 6 [6.98%], medication group: *n* = 7 [8.33%]). The main reasons for dropout were time restrictions, change in residential location, dissatisfaction with the treatment, and fear of needling (Supplementary Table 12 in Supplementary material 2). We did not lose contact with any of the participants during the follow-up phase. Details of the trial procedure are presented in Figure 1 in accordance with the Standards For Reporting Interventions In Controlled Trials Of Acupuncture guidelines (MacPherson et al., 2002).

The participants in acupuncture group received an average of 22.95 sessions of treatment, whereas those in the medication group received an average of 22.71 treatment sessions. Regarding the degree of participation, 93.02% of the participants in the acupuncture group and 91.67% of those in the medication group received at least 20 (≥80%) of the planned acupuncture treatment sessions. By tablet count, 93.02% of the participants in the acupuncture group and 91.67% of those in the medication group achieved at least 80% of the planned medication adherence.

The demographic characteristics and baseline comparable of the ITT population are summarized in Table 1. The mean age of the participants was 35.81 years old, with 53.53% of the patients being > 35 years old. The mean menstrual cycle length and mean menstruation length were 28.99 and 5.51 days, respectively. Migraine history was well balanced, with no clinically relevant differences between the two groups. The average number of migraine days during and outside the perimenstrual period over the three-cycle baseline phase were 1.75 and 1.94, respectively; approximately 67.65% of the participants used acute pain medication during this period. The baseline characteristics of per-protocol (PP) population were similar to those of the ITT population (Supplementary Table 5 in Supplementary material 2).

**TABLE 1:** Demographic and baseline characteristics of the 170 patients included in the intention-to-treat analysis.

Characteristics	Acupuncture group (*n* = 86)	Medication group (*n* = 84)	Total (*n* = 170)
Mean (SD) age, y	36.44 (6.74)	35.15 (6.73)	35.81 (6.75)
Mean (SD) duration from the time of migraine diagnosis to baseline, y	8.24 (7.06)	8.35 (5.72)	8.30 (6.42)
Family history, n (%)	28 (32.56)	27 (32.14)	55 (32.35)
Accompanying symptoms			
Nausea or vomiting, n (%)	82 (95.35)	80 (95.24)	162 (95.29)
Photophobia or phonophobia, n (%)	57 (66.28)	52 (61.90)	109 (64.12)
Others, n (%)	22 (25.58)	15 (17.86)	37 (21.76)
Dysmenorrhea, n (%)	28 (32.56)	21 (25.00)	49 (28.82)
Mean (SD) length of menstrual cycle, day	29.24 (2.32)	28.74 (2.15)	28.99 (2.24)
Mean (SD) length of menstruation, day	5.53 (1.15)	5.49 (1.19)	5.51 (1.16)
Mean (SD) number of migraine days during the perimenstrual period	1.83 (0.73)	1.67 (0.55)	1.75 (0.65)
Mean (SD) number of migraine days outside the perimenstrual period	2.07 (1.43)	1.81 (0.99)	1.94 (1.23)
Mean (SD) number of migraine hours during the perimenstrual period	21.03 (12.25)	19.77 (11.18)	20.41 (11.71)
Mean (SD) number of migraine hours outside the perimenstrual period	14.72 (8.85)	14.15 (8.27)	14.44 (8.55)
Mean (SD) pain VAS score recorded during the perimenstrual period	7.30 (1.45)	7.27 (1.41)	7.28 (1.43)
Mean (SD) pain VAS score recorded outside the perimenstrual period	6.13 (1.45)	6.04 (1.48)	6.08 (1.46)
Use of acute pain medication, n (%)	60 (69.77)	55 (65.48)	115 (67.65)

SD, standard deviation; VAS, visual analog scale.

### Efficacy findings

#### Primary outcome measure

**FIGURE 2:**
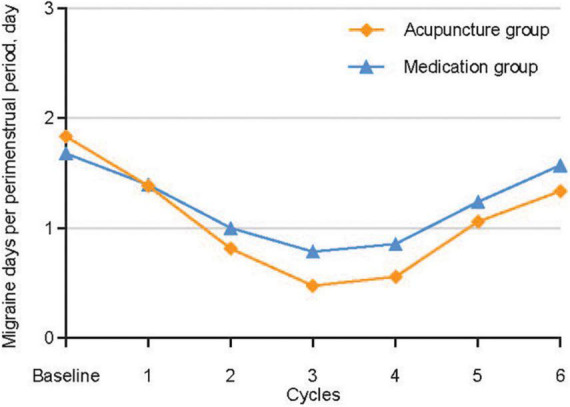
Primary outcomes throughout the trial.

Figure 2 and Table 2 show the primary analysis performed using ITT data. The average number of migraine days recorded per perimenstrual period was 1.83 (95% confidence interval [CI], 1.68–1.99) at baseline and 0.89 (95% CI, 0.74–1.05) over cycles 1−3 in the acupuncture group, and 1.67 (95% CI, 1.55–1.79) at baseline and 1.06 (95% CI, 0.95–1.17) over cycles 1−3 in the medication group. The reduction from the baseline average number of migraine days per perimenstrual period over cycles 1−3 was 0.94 (95% CI, 0.82–1.07) in the acupuncture group and 0.61 (95% CI, 0.50–0.71) in the medication group, with a 0.33 between-group difference that favored the acupuncture group (95% CI, 0.17–0.50; *P* < 0.001) (Table 2). Similar between-group differences were observed in the PP population (Supplementary Table 6 in Supplementary material 2), the sensitivity analysis performed using a multiple imputation method with missing data (Supplementary Table 4 in Supplementary material 2), and the sensitivity analysis performed for controlling for individual menstrual cycle lengths in the mixed-effects model (Supplementary Appendix 3 and Supplementary Tables 8–11 in Supplementary material 2).

**TABLE 2:** Primary and secondary outcomes (intention-to-treat population)^a^.

Outcome	Acupuncture group (*n* = 86)	Medication group (*n* = 84)	Between-group difference	
			Value (95% CI)	*P*-value^b^
**Primary outcome**				
Average number of migraine days per perimenstrual period^c^, cycles 1−3, mean (95% CI)				
	0.89 (0.74 to 1.05)	1.06 (0.95 to 1.17)	0.17 (−0.24 to 0.36)	0.0862
Change from the baseline average number of migraine days per perimenstrual period^d^, cycles 1−3, mean (95% CI)^e^				
	0.94 (0.82 to 1.07)	0.61 (0.50 to 0.71)	0.33 (0.17 to 0.50)	0.0001
**Secondary outcomes**				
Change from the baseline average number of migraine days per perimenstrual period, cycles 4−6, mean (95% CI)^e^				
	0.84 (0.72 to 0.97)	0.44 (0.32 to 0.56)	0.41(0.24 to 0.58)	<0.0001
Change from the baseline average number of migraine days outside the perimenstrual period, mean (95% CI)^e^				
Cycles 1−3	1.08 (0.89 to 1.27)	0.49 (0.39 to 0.60)	0.59 (0.37 to 0.80)	<0.0001
Cycles 4−6	0.95 (0.75 to 1.16)	0.31 (0.21 to 0.41)	0.64 (0.41 to 0.87)	<0.0001
Change from the baseline mean number of migraine hours during the perimenstrual period^f^, mean (95% CI)^e^				
Cycles 1−3	7.61 (6.22 to 9.02)	5.01 (3.83 to 6.20)	2.60 (0.79 to 4.41)	0.0055
Cycles 4−6	5.65 (4.36 to 6.93)	3.09 (2.14 to 4.05)	2.55 (0.97 to 4.13)	0.0019
Change from the baseline mean number of migraine hours outside the perimenstrual period^f^, mean (95% CI)^e^				
Cycles 1−3	4.56 (3.56 to 5.66)	2.59 (1.99 to 3.19)	1.97 (0.82 to 3.12)	0.0010
Cycles 4−6	3.52 (2.66 to 4.37)	1.51 (0.86 to 2.15)	2.01 (0.95 to 3.07)	0.0003
Change from the baseline mean VAS score during the perimenstrual period, mean (95% CI)^e^				
Cycles 1−3	2.52 (2.15 to 2.90)	2.14 (1.84 to 2.44)	0.38 (−0.10 to 0.85)	0.1221
Cycles 4−6	2.39 (2.05 to 2.73)	1.30 (1.09 to 1.52)	1.08 (0.69 to 1.48)	<0.0001
Change from the baseline mean VAS score outside the perimenstrual period, mean (95% CI)^e^				
Cycles 1−3	2.17 (1.76 to 2.57)	1.81 (1.53 to 2.09)	0.36 (−0.12 to 0.85)	0.1462
Cycles 4−6	1.45 (1.20 to 1.69)	0.95 (0.71 to 1.18)	0.50 (0.17 to 0.83)	0.0039
50% migraine responder rate, participants, n (%)^g^				
Cycles 1−3	54 (62.8%)	36 (42.9%)	19.9 (5.22 to 34.64)	0.0092
Cycles 4−6	50 (58.1%)	32 (38.1%)	20.0 (5.33 to 34.76)	0.0089
Use of acute pain medication, participants, n (%)^g^				
Cycles 1−3	24 (27.9%)	35 (41.7%)	13.8 (−27.94 to 0.42)	0.0595
Cycles 4−6	40 (46.5%)	46 (54.8%)	8.3 (−23.23 to 6.73)	0.2821

^a^Six participants in the acupuncture group and seven in the medication group were missing information on the average number of migraine days during/outside the perimenstrual period, mean number of migraine hours during/outside the perimenstrual period, and mean VAS score during/outside the perimenstrual period over cycles 1−3. Six participants in the acupuncture group and seven in the medication group were missing these details in cycles 4−6. The missing data of participants who dropped out were replaced using the last observation carried forward method. The number of participants with imputed data: 6 (7.0%) in the acupuncture group and 7 (8.3%) in the medication group.

^b^All tests were two-sided. *P*-value < 0.05 was considered significant.

^c^The perimenstrual period is starts from the two days before the onset of menstruation to first three days of menstruation.

^d^Baseline was calculated as the cycle average of the three-cycle screening phase prior to the start of treatment.

^e^Analyzed by fitting a mixed-effect model using the baseline value as a covariate, treatment as a fixed effect, and center as a random effect.

^f^Baseline was calculated the daily average of the three-cycle screening phase prior to the start of treatment.

^g^Analyzed using the chi-square test.

CI, confidence interval; VAS, visual analog scale.

#### Key secondary outcome measures

The acupuncture group achieved a significantly greater decrease in the average number of migraine days outside each perimenstrual period and the mean number of migraine hours during and outside the perimenstrual period over cycles 1−3 and 4−6 than the medication group. The between-group difference in the reduction of the average number of migraine days outside the perimenstrual period was 0.59 (95% CI, 0.37–0.80; *P* < 0.001) for cycles 1−3, and 0.64 (95% CI, 0.41–0.87; *P* < 0.001) for cycles 4−6. The between-group difference in the reduction of the mean number of migraine hours during the perimenstrual period was 2.60 (95% CI, 0.79–4.41; *P* = 0.006) for cycles 1−3, and 2.55 (95% CI, 0.97–4.13; *P* = 0.002) for cycles 4−6. The between-group difference in the reduction of the mean number of migraine hours outside the perimenstrual period was 1.97 (95% CI, 0.82–3.12; *P* = 0.001) for cycles 1−3, and 2.01 (95% CI, 0.95–3.07; *P* < 0.001) for cycles 4−6.

The acupuncture group showed greater decrease in the mean VAS score recorded during and outside the perimenstrual period over cycles 4−6 (not over cycles 1−3) than the medication group (VAS score during the perimenstrual period: between-group difference, 0.38) (95% CI, −0.10–0.85, *P* = 0.12); VAS score outside the perimenstrual period: between-group difference, 0.36 (95% CI, −0.12–0.85, *P* = 0.15). The between-group difference in the reduction of the mean VAS score during and outside the perimenstrual period was 1.08 (95% CI, 0.69–1.48; *P* < 0.001) and 0.50 (95% CI, 0.17–0.83; *P* = 0.004), respectively, for cycles 4−6.

The ≥50% migraine responder rates in the acupuncture and medication groups were 62.8% and 42.9% at the end of the treatment phase, and 58.1% and 38.1% at the end of follow-up phase, respectively. (Table 2). The between-group difference in the responder rates for the treatment and the follow-up phases were 19.9% (95% CI, 5.22–34.64; *P* = 0.009) and 20.0% (95% CI, 5.33–34.76; *P* = 0.009), respectively. Data from the acupuncture group demonstrated that ≥50% migraine responder rates were sustained throughout the six-cycle interval.

Changes from the baseline mean VAS score during and outside the perimenstrual period over cycles 1−3 (*P* > 0.05 for all) did not differ between the two groups. We did not note any between-group difference in the proportion of participants that used acute pain medication during cycles 1−3 and 4−6 (*P* > 0.05).

### Safety

A total of 22 participants (12.94%) experienced at least one treatment-emergent adverse event (TEAE), but none of them required special medical interventions. Eight (9.30%) participants in the acupuncture group and 14 (16.67%) in the medication group had TEAEs, which mainly included dermorrhagia, numbness, and lassitude for acupuncture-related AEs, and nausea, palpitations, dyspepsia, upper abdominal pain, heartburn, somnolence, dizziness, and sweating attack for medication-related AEs (Table 3). These events were mild or moderate. The incidence of TEAEs was generally balanced between the acupuncture and medication groups (*P* = 0.15).

**TABLE 3:** Treatment-emergent adverse events (safety population)^a^.

Adverse events	Acupuncture group (*n* = 86)		Medication group (*n* = 84^b^)	
	Participants, N (%)	Events, N	Participants, N(%)	Events, N
Total	8 (9.30%)	8	14 (16.67%)	16
Related to acupuncture overall	3 (3.49%)^c^	3	4 (4.76%)^d^	4
Dermorrhagia	3 (3.49%)	3	2 (2.38%)	2
Numbness	0	0	1 (1.19%)	1
Lassitude	0	0	1 (1.19%)	1
Related to medication overall	5 (5.81%)^e^	5	10 (11.90%)^f^	12
Nausea	0	0	2 (2.38%)	2
Palpitations	0	0	2 (2.38%)	2
Dyspepsia	2 (2.33%)	2	1 (1.19%)	2
Upper abdominal pain	1 (1.16%)	1	1 (1.19%)	1
Heartburn	0	0	1 (1.19%)	2
Somnolence	1 (1.16%)	1	1 (1.19%)	1
Dizziness	0	0	1 (1.19%)	1
Sweating attack	1 (1.16%)	1	1 (1.19%)	1

^a^Adverse events were analyzed in all participants who received at least one session of treatment. Adverse events experienced by the same participant were counted according to type rather than frequency. Adverse events of different types experienced by a single participant were defined as independent adverse events. Multiple occurrences of an adverse event in a single participant was defined as one adverse event.

^b^Two participants in the medication group did not receive treatment.

^c^Adverse events related to true acupuncture.

^d^Adverse events related to sham acupuncture.

^e^Adverse events related to placebo naproxen.

^f^Adverse events related to naproxen.

In the acupuncture group, three participants (*n* = 3 [3.49%]) reported dermorrhagia (without formation of subcutaneous hematoma) at a few acupoints after needle removal. These AEs were classified as a cause relevant to acupuncture. Five participants reported gastrointestinal, central nervous system, and dermatologic events (dyspepsia: *n* = 2 [2.33%]; upper abdominal pain: *n* = 1 [1.16%]; somnolence: *n* = 1 [1.16%]; sweating attack: *n* = 1 [1.16%]). These AEs were classified as a cause relevant to placebo medication. In the medication group, 10 participants reported gastrointestinal, cardiovascular, central nervous system, and dermatologic events (nausea: *n* = 2 [2.38%]; palpitations: *n* = 2 [2.38%]; dyspepsia: *n* = 1 [1.19%]; upper abdominal pain: *n* = 1 [1.19%]; heartburn: *n* = 1 [1.19%]; somnolence: *n* = 1 [1.19%]; dizziness: *n* = 1 [1.19%]; sweating attack: *n* = 1 [1.19%]). These AEs were considered to be related to the side effects of naproxen. Four participants reported acupuncture-related AEs (dermorrhagia: *n* = 2 [2.38%]; numbness: *n* = 1 [1.19%]; lassitude: *n* = 1 [1.19%]). The participants who reported these AEs fully recovered and continued the trial.

### Credibility of blinding

At cycle 1.5, 61 of 80 participants in the acupuncture group and 53 of 77 participants in the medication group guessed their treatment to be true acupuncture plus placebo naproxen (kappa coefficient: 0.075 [−0.07, 0.22]; *P* = 0.30). At cycle 3, the numbers were 62/80 participants in the acupuncture group and 49/77 participants in the medication group (kappa coefficient: 0.14 [−0.00, 0.28]; *P* = 0.06) (Table 4). There was no significant between-group difference in the proportion of participants who guessed “true acupuncture plus placebo naproxen” when asked if they received true acupuncture plus placebo naproxen or sham acupuncture plus naproxen treatment at cycles 1.5 and 3.

**TABLE 4:** Credibility of blinding.

	Treatment guess, No. (%)	Acupuncture group (*n* = 80^a^)	Medication group (*n* = 77^b^)	Kappa Coefficient (95% CI)	*P* ^c^
Cycle 1.5	True acupuncture plus placebo naproxen	61 (76.2%)	53 (68.8%)	0.075 (−0.07, 0.22)	0.30
	Sham acupuncture plus naproxen	19 (23.8%)	24 (31.2%)		
Cycle 3	True acupuncture plus placebo naproxen	62 (77.5)	49 (63.6%)	0.14 (−0.00, 0.28)	0.06
	Sham acupuncture plus naproxen	18 (22.5%)	28 (36.4%)		

^a^Six participants in the acupuncture group were not recorded at Cycles 1.5 and 3 because they dropped out.

^b^Seven participants in the medication group were not recorded at Cycles 1.5 and 3 because they dropped out.

^c^*P* was calculated from a kappa analysis.

CI, confidence interval.

## Discussion

This double-dummy, randomized, controlled trial was conducted to evaluate the efficacy of acupuncture as a preventive treatment for MRM. The results indicated that the acupuncture group showed significantly greater reductions in the number of migraine days and hours from baseline and greater ≥50% migraine responder rate than the medication group during the treatment (cycles 1−3) and follow-up (cycles 4−6) phases. Moreover, the acupuncture group reported relatively better alleviation of headache intensity during the follow-up phase than the medication group, but not during the treatment phase. However, the proportion of participants who used acute pain medication did not differ between the two groups.

The reduction in the average number of migraine days per perimenstrual period in the acupuncture group was 0.94 for cycles 1−3 and 0.84 for cycles 4−6. Both results were greater than the minimal clinically important difference (MCID) of 0.5 days (National Clinical Guideline Centre, 2012). Nevertheless, the between-group difference in these reductions was 0.33 days for cycles 1−3 and 0.41 days for cycles 4−6, which do not meet the MCID. This demonstrates that the difference in the reduction of the number of migraine days between the acupuncture and medication groups may be statistically significant but does not indicate a clinically meaningful difference in the improvement of migraine frequency during the perimenstrual period.

The sensitivity analyses of the PP population (between-group difference: 0.37 days) (Supplementary Table 6 in Supplementary material 2), conducted using a multiple imputation method with missing data (between-group difference: 0.35 days) (Supplementary Table 4 in Supplementary material 2) and controlling for individual variations in menstrual cycle length (between-group difference: 0.33 days) (Supplementary Table 11 in Supplementary material 2), were similar to the ITT analysis performed using the LOCF method. The between-group differences in the reduction of the average number of migraine days outside the perimenstrual period over cycles 1−3 and cycles 4−6 were greater than the MCID of 0.5 days. This suggests that acupuncture may have a statistically significant and clinically meaningful preventive effect by reducing the frequency of migraine outside the perimenstrual period over six cycles. Our findings are inconsistent with those of a previous study, which indicated that there were no significant differences between the treatment (*n* = 15) and control (*n* = 13) groups during treatment or follow-up 3 and 6 months later (Linde M. et al., 2005). This variation in findings may be associated with differences in the number of participants included and in the acupoints chosen in both studies. The total number of participants included in the present study was 172, whereas only 28 participants were included in the former one study by Linde et al.; thus, it was easier for them to get non-significant results. Besides, according to the theories of traditional Chinese medicine, the efficacy of acupuncture intervention is affected by the acupoints used. The acupoints used in the present study and in the former study are different, partially accounting for the discrepancy between the results of the two studies.

Visual analog scale score is widely accepted as one of the major measures of headache intensity. In this trial, there was no significant difference between the VAS scores of the acupuncture and the medication groups. However, over cycles 4−6, the VAS score recorded during and outside the perimenstrual period reduced by 2.39 and 1.45 points, respectively, in the acupuncture group, compared with a reduction of 1.30 or 0.95, respectively, in the medication group, with a between-group difference of 1.08 and 0.50 points (VAS during the perimenstrual period, *P* < 0.0001; VAS outside the perimenstrual period, *p* = 0.0039). In the study by Linde M. et al., 2005, the reduction of VAS score at week 24 was 1.0 for women who received acupuncture, and −0.2 for those who received sham acupuncture (*P*>0.05). The sample size of the present study differs from that of the study by Linde et al. (*n* = 172 vs. 28). Consequently, the differences between the VAS scores of the treatment and control groups in the two studies are probably comparable and are not clinically meaningful. A mean reduction in baseline VAS score of 3.5 points 2 hours after the intake of medication for moderate and severe headache has been reported as the MCID (Aicher et al., 2012). Similar to the results of the average number of migraine days per perimenstrual period, the reductions of VAS scores over cycles 4−6 suggest that acupuncture was more effective than medication in reducing the intensity of headache; however, the between-group difference was smaller than the MCID. Although the pain reduction reported by the two groups during the treatment phase was not significantly different, the results indicated that patients in the acupuncture group achieved greater pain reduction during the follow-up phase than those in the medication group. Such results indicate that acupuncture yielded a long-term therapeutic effect.

To a certain extent, we did not observe any difference between the acupuncture and medication groups in terms of the proportion of participants that used acute pain medication. However, the between-group difference in the reduction of the number of migraine days during and outside the perimenstrual period was statistically significant. Since we recorded the proportion of participants who used acute pain medication, but not the dose of medication used, it was not feasible to compare actual medication usage between the groups. This should be considered in future research.

Interestingly, although changes from the baseline number of migraine days and VAS score decreased over time (baseline, cycle 3, and cycle 6) in both the acupuncture and medication groups, the between-group differences in the changes during the perimenstrual period became stronger over time. The abovementioned results may be caused by a markedly decreased tendency to changes from the baseline number of migraines days and VAS score in the medication group. Although these results share similarities with those of the former one, there are some differences between them. In the study by Linde M. et al., 2005, the number of migraine days decreased after 12 weeks of acupuncture treatment and 12 weeks of follow up, but stayed relatively the same at 36 weeks. These discrepancies may be caused by variations in the duration of follow-up, sham acupuncture design, and number of acupuncture sessions.

In present study, the sham acupuncture method was performed by choosing non-disease-related acupoints; however, the stimulation experienced by the patient was same as that induced by real acupuncture. Present studies have focused on the effects due to acupoint specificity. Repetitive results of Yang et al. reported the different mode of activated brain metabolism within the disease-related acupoint and non-disease-related acupoint groups, convincing a superiority of acupoint specificity in pain relieving (Yang et al., 2012, 2014). Thus, this type of sham acupuncture has been found to have more therapeutic effects than non-penetrating sham acupuncture and is considered not totally inert (MacPherson et al., 2014) due to the physiological effect it produces. The activation of ergoreceptors, which deliver the information in A-delta or type II or III afferents into the spinal cord, leads to the afferent stimulation produced by acupuncture (Andersson and Lundeberg, 1995; Hui et al., 2000). Moreover, the activities of acupuncture are associated with the sensory and affective components of pain through the activation of descending pain-inhibiting pathways and the deactivation of the limbic structures, respectively (Han, 2003; Hui et al., 2005). Recently, several studies have demonstrated that even a light touch on the skin can trigger activity in the insular region, but not in the somatosensory cortex, by stimulating mechanoreceptors coupled to slow-conducting unmyelinated (C) afferents, which induces a “limbic touch” response, leading to the emotional and hormonal reactions commonly seen following caressing. Therefore, as neither the sham acupuncture method used in the present study nor non-penetrating sham acupuncture are totally inert, they cannot be considered “real placebo” because they activate C tactile afferents, resulting in the alleviation of unpleasantness and the re-establishment of the patient’s sense of self-esteem and wellbeing (Damasio, 1999; Olausson et al., 2002; Mohr et al., 2005). The application of blunt needles on effective acupoints was not used in this trial as a control intervention because patients in China are very familiar with the sensations caused by acupuncture and may easily identify the sensation caused by blunt needles. Most physiological mechanisms proposed for acupuncture may activate unmyelinated afferent nerves, which can influence pain perception, as mentioned above (Lund and Lundeberg, 2006). Meanwhile, different types of placebos may have different placebo effects (Finniss et al., 2010). Acupuncture−with its repeated sessions, intense provider contact, slightly painful procedure, an often “exotic” model of symptom explanation, and associated relaxation during sessions−may maximize such placebo effects (Linde et al., 2016). Sham acupuncture is associated with greater placebo effects than a placebo pill or other non-pharmacological sham interventions (Oken, 2008; Zhang et al., 2008). Therefore, the placebo effects of placebo medication and sham acupuncture on participants may be different. However, this difference is less likely to cause between-group differences than the real difference between acupuncture and medication.

Generalizability of the results of this trial may be limited by the following factors: low baseline number of migraine days (mean baseline number of migraine days during the perimenstrual period: 1.83 and 1.67 days in the acupuncture and medication groups, respectively; mean baseline number or migraine days outside the perimenstrual period: 2.07 and 1.81 days in the acupuncture and medication groups, respectively), limited and monoethnic study population (all Chinese), lack of assessments of the participants’ expectations (Mao et al., 2007) at baseline and treatment satisfaction (Trutnovsky et al., 2018) at cycles 3 and 6, lack of medication usage records, and lack of evaluation of other quality of life outcomes, e.g., the Migraine-Specific Quality of Life Questionnaire score (Cole et al., 2007) and the Migraine Disability Assessment Score (Stewart et al., 2001). More appropriate sham acupuncture, such as non-penetrating needles, should be used for a minimal therapeutic effect as high-qualified sham control. In addition, this study was supposed to be a 2 × 2 design with four groups (true acupuncture plus naproxen, true acupuncture plus placebo, sham acupuncture plus naproxen, and sham acupuncture plus placebo) had sufficient funding support and time been available.

## Conclusion

In this study, participants with MRM who received a three-cycle acupuncture treatment experienced significantly greater reductions in the number of migraine days over six cycles than those who received medication, indicating that acupuncture is effective for preventing MRM and has a sustained treatment effect over six cycles. Considering these promising results, it indicates that MRM patients with an inter-individual difference in drug response or those who are unwilling to accept possible drug-induced adverse events may benefit from acupuncture treatment. Consequently, acupuncture as a complementary and alternative medicine therapy can be a more potent and safe treatment for MRM prophylaxis.

## Data availability statement

The raw data supporting the conclusions of this article will be made available by the authors, without undue reservation.

## Ethics statement

The studies involving human participants were reviewed and approved by the Research Ethical Committee of Beijing Hospital of Traditional Chinese Medicine in Beijing. The patients/participants provided their written informed consent to participate in this study.

## Author contributions

L-PW and H-LL designed the study protocol. L-PW, X-HJ, and BL conceived and designed the study and revised the manuscript for intellectual content. T-LL, L-PZ, M-NW, Z-YQ, L-MN, JG, X-ZZ, and Y-HL had an effective role in the implementation of this study and the data collection. LL, C-SZ, FH, and LZ performed the statistical analyses and prepared the figures and tables. LL wrote the manuscript. K-LW contributed to a thorough revision of the manuscript. All authors revised and approved the final manuscript.

## Abbreviations

AE: adverse events
CI: confidence interval
ITT: intention-to-treat
ICHD: International Classification of Headache Disorders
LOCF: last observation carried forward
MCID: minimal clinically important difference
MRM: menstruation-related migraine
PP: per-protocol
VAS: visual analog scale.
