# Supplementary material for: Acupuncture for menstruation-related migraine prophylaxis: A multicenter randomized controlled trial
Source: Front Neurosci. 2022 Aug 26;16:992577. doi: 10.3389/fnins.2022.992577 (PMC9459087; doi:10.3389/fnins.2022.992577)
Supplement: Supplementary file 1 [file Data_Sheet_1.pdf]

Supplemental Material 1

This supplement contains the following items:

1. Final protocol, summary of changes from the published original protocol.

2. Final statistical analysis plan, summary of changes from the published original protocol.

Acupuncture as prophylaxis for menstrual-related migraine: study protocol for a multicenter randomized controlled trial

Clinical centers:

1. Beijing Hospital of Traditional Chinese Medicine, Capital Medical University
2. Peking University Third Hospital
3. Beijing Tiantan Hospital, Capital Medical University
4. Xiyuan Hospital, China Academy of Chinese Medical Sciences

Data Management and Statistical Centers:

Research Centre of Clinical Epidemiology, Peking University Third Hospital, Beijing, China

Data:

Original protocol date: November 29, 2012

Amendment date: April 25, 2013

Table of Contents

1. Study Contact and Organization 4
   1.1 Study Contacts 4
   1.2 Recruiting Centers 4
   1.3 Collaborating Centers 5
2. Study Design 6
   2.1 Study Overview 6
   2.2 Background 6
   2.3 Study Objectives and Hypothesis 8
   2.4 Methodology 8
      2.4.1 Trial design 8
      2.4.2 Subjects 9
      2.4.3 Trial flow Chart 11
      2.4.4 Outcomes Measurements 14
3. Safety Assessment 17
4. Interventions 17
   4.1 Treatment of the acupuncture group 18
   4.2 Treatment of medication group 19
   4.3 Permitted and prohibited concomitant treatments 20
5. Informed Consent 20
6. Quality Control 26
7. Data Management 27
   7.1 The Raw Data Management and Archiving 27
   7.2 Data Entry and Storage 27
      7.2.1 Database Building and Testing, Data Entry Interface 27
      7.2.2 Data Entry and Inspection 28
   7.3 Data Verification and Problems Solving 28
   7.4 Medical Coding 28
   7.5 Data Blinding Review and Data Management Report 28
   7.6 Database Locking 29
8. Statistical consideration 29
   8.1 Statistical Analysis 29
   8.2 Statistical Analysis Plan (SAP) 30
9. Ethical principle 31
10. Funding 31
11. Update on the Published Protocol 31

1. Study Contact and Organization

1.1 Study Contacts

<u>Principal Investigator for grant and trial</u>

Linpeng Wang, MD

Beijing Hospital of Traditional Chinese Medicine, Capital Medical University, Beijing Key Laboratory of Acupuncture Neuromodulation, Beijing, China

23 Meishuguanhou Street, Dongcheng District, Beijing, China, 100010

Phone: +86 13911406703

Email: wlp5558@sina.com

1.2 Recruiting Centers

<u>Beijing Hospital of Traditional Chinese Medicine, Capital Medical University</u>

Huilin Liu, MD PhD

Center Principal Investigator, Department of Acupuncture and moxibustion

23 Meishuguanhou Street, Dongcheng District, Beijing, China, 100010

Phone: +86 13681395789

Email: lhlxwy@aliyun.com

<u>Peking University Third Hospital</u>

Jia Guo, MD

Center Principal Investigator, Traditional Chinese Medicine Department

49 Huayuan North Street, Haidian District, Beijing, China, 100191

Phone: +86 13611223675

Email: guojia65@163.com

<u>Beijing Tiantan Hospital, Capital Medical University</u>

Xiaozhe Zhang, MD

Center Principal Investigator, Department of Pain Management

119 South Fourth Ring West Street, Fengtai District, Beijing, China,100070

Phone: +86 13683115750

Email: zhangxiaozhezxz@126.com

Xiyuan Hospital, China Academy of Chinese Medical Sciences

Yonghui Lu, MD

Center Principal Investigator, Acupuncture and Moxibustion Department

1 Xiyuancaochang, Haidian District, Beijing, China,100091

Phone: +86 13521776025

Email: yhlu2008@sina.com

1.3 Collaborating Centers

Research Center of Clinical Epidemiology, Peking University Third Hospital

Lin Zeng, PhD

Phone: +86 15611963082

Email: zlwhy@163.com

Institute of Acupuncture and Moxibustion, China Academy of Chinese Medical Sciences

Xianghong Jing, MD PhD

16 Nanxiaojie Dongzhimennei, Dongcheng, Beijing, China, 100700

Phone: +86 13671120972

Email: jxhtjb@263.net

School of Health and Biomedical Sciences, RMIT University

Claire Shuiqing Zhang, MD PhD

Plenty Road, Bundoora VIC 3083, Australia

Phone: +61 3 9925 7002

Email: claire.zhang@rmit.edu.au

Centre for Sensory-Motor Interaction, Department of Health Science & Technology, Aalborg University

Kelun Wang, MD PhD

Fredrik Bajers Vej 7-D3 DK-9220 Aalborg East,Denmark

Phone: +45 5014 7630

Email: kelun@hst.aau.dk

School of Information Management, Wuhan University

Fan He, PhD

299 Bayi Stret, Wuchang District, Wuhan, China, 430072

Phone: +86 15172369886

Email: marco_hefan@foxmail.com

2. Study Design

2.1 Study Overview

The objective of this study was to assess the efficacy of acupuncture in the preventive treatment of MRM.

2.2 Background

Migraine is a common neurological disorder. Population based studies suggest that 6% to 7% of men and 15% to 18% of women experience migraine (1, 2). More than 50% of female patients report that their migraines are associated with the menses(3). Attack onset is usually before the age of 20 years, with peak prevalence between the ages of 25 and 55 years, declining with menopause(4-6). Increasing evidence links menstrual migraine to the female sex hormones(3, 7).

Approximately 14% of female migraineurs have migraine only during menstruation (pure menstrual migraine (PMM)) while 60% suffer from migraine at both menses and other times during the menstrual cycle (menstrual-related migraine (MRM)) (8). Most of the data in the literature report that MRM causes significant limitations of daily activities (for example, nausea, vomiting, and photo-phonophobia), and the attacks are generally longer, more severe, and less drug-responsive than non-menstrual ones(9, 10).

Given the particular clinical picture that characterizes MRM, it is not surprising that the attacks are difficult to treat. The options available for the treatment of MRM include acute therapy and prophylaxis (short-term preventive therapy and long-term preventive therapy) (11-

13). Acute therapy is initiated first. As MRM is unique in its predictability, treatment can be targeted to the period of time when patients are most likely to experience migraine. The treatment window for menstrual migraine occurs between 2 days before and 3 days after the onset of menses(12). The goals of prophylactic strategies are to reduce attack frequency, severity and duration, improve responsiveness of treatment for acute attacks, improve function, and reduce disability. Prophylactic medications include non-steroidal anti-inflammatory drugs (NSAIDs), triptans, estrogen, magnesium, dihydroergotamine (DHE), methysergide, and vitamin E(13). Caution should be taken when the drug is used within the same month for treating migraine attacks occurring during perimenstrual period and outside perimenstrual period due to the risk of medication-overuse headache, a risk which is, however, common to all medications used for menstrual-migraine prophylaxis or treatment(13).

Acupuncture, which is one of the main treatment modalities of Traditional Chinese Medicine (TCM), has been used for both the prevention and treatment of diseases for over 2,000 years. Several studies (14-17) have already reported the encouraging results in the therapy for migraine by acupuncture. A German randomized controlled trial (n = 794) showed that 11 acupuncture treatments given within 6 weeks were at least as effective as a β-blocker taken daily over 6 months(14). Yang et al. found that acupuncture treatment was more effective and safer than topirmarate (15). Wang and co-authors reported that acupuncture was more effective than flunarizine in decreasing the duration of migraine attacks(16). Therefore, acupuncture should be considered as an option for patients with migraine(17). Although the physiological mechanism is still unclear (18), in the last 40 years, many theoretically plausible models have been developed, such as the principle of counter-irritation diffuse noxious inhibitory control (DNIC) and the endorphin hypothesis Melzack's gate control theory (19-21). DNIC is regarded as a possible factor explaining central pain modulation through acupuncture (22). The endorphin hypothesis serves to explain a short-term analgesic effect of acupuncture, which is considered very robust.

However, based on a review of published literatures, the guidelines did not make treatment recommendation for acupuncture, because this therapy lacked adequate randomized controlled trials to support its effect. When reviewing the evidence of acupuncture for MRM, only one

published randomized controlled trial could be found(22). The objectives of that study were to introduce a new method for controlled trials of acupuncture for headache and to examine the role of needling per se. No significant differences were found between the verum group and the placebo group.

In this study, we will perform a randomized controlled trial to investigate the efficacy of acupuncture treatment as prophylaxis for MRM.

2.3 Study Objectives and Hypothesis

The objective of this study is to assess the efficacy of acupuncture as the preventive treatment for women with MRM.

We hypothesize that acupuncture is different from medication in decreasing migraine days for women with MRM.

2.4 Methodology

2.4.1 Trial design

This is a multicenter, double-dummy, participant-blinded, randomized controlled clinical trial at 4 centers in China.

2.4.1.1 Randomization

This study, we will use blocks randomization, stratified according to center. A central randomization system will be applied in our trial. The randomizing scheme will be produced by staff of the Research Center of Clinical Epidemiology affiliated to Peking University using statistical analysis software SAS9.4 with “proc plan” program. After production, it will be signed and sealed by the staff who produce it and keep by other staff who take no part in this trial. It will not be allowed to be checked by anyone except the top system administrator. Acupuncturists in each center will be responsible for getting random numbers. Via inputting the participants’ sex and birthday in the central randomization system through the phone or the web, they will get the random number.

2.4.1.2 Blinding

In this study, participants, outcome assessors and statisticians will be blinded to treatment allocation. To ensure all participants being blinded to their group allocation, the placebo naproxen will be prepared by Diao Group Chengdu Pharmaceutical LTD (Chengdu, China) with identical taste, smell and appearance as true naproxen, and the sham acupuncture will produce same stimulation as true acupuncture. Participants of different groups will be treated separately and blinded to which acupuncture method they would receive. For blinding assessment, all participants will be requested to guess whether they received 'True acupuncture plus placebo naproxen' or 'Sham acupuncture plus naproxen' within five minutes after their treatments at cycles 1.5 and 3.

2.4.1.3 Sample Size

According to the previous pilot study(23), we anticipated that the number of migraine days over 12 weeks would be 3.1(SD 2.7) days in the acupuncture group and 5.2 (SD 4.4) in the medication group. With a 2-sided significance level of 5% and power of 90%, 68 participants for each group would be required, as calculated by PASS 2008 software (NCSS, Kaysville, Utah, USA). With an estimated loss-to-follow-up rate of 20%, we planned to enroll a total of 172 participants with 86 participants per group.

2.4.2 Subjects

2.4.2.1 Eligibility criteria

Women will be included in the study if they met the following criteria. (1) Diagnosis of MRM(24): MRM without aura (code A1.1.2), in which migraine without aura always occurs on days 1 ± 2 of menstruation in at least two out of three menstrual cycles, and at other times due to different triggering factors or for no apparent specific reason (International Classification of Headache Disorders - second edition [ICHD-II]); (2) Regular menstrual cycle (25-35 days); (3) could predict within three days both the onset of menstruation and perimenstrual migraine attacks; (4) Repeated migraine attacks, frequency of nonmenstrual migraine is more than once

a month; (5) Written informed consent.

2.4.2.2 Exclusion criteria

Women will be excluded from the study if they met the following criteria. (1) Chronic migraine, tension-type headache, cluster headache, and other primary headaches; (2) Secondary headache and other neurological diseases;(3) Relatively severe systemic diseases (cardiovascular disease, acute infectious disease, hematopathy, endocrinopathy, and allergy); (4) Headache caused by otorhinolaryngology diseases or intracranial pathological changes; (5) Oral contraceptives, pregnancy, or lactation period; (6) Use of prophylactic migraine medication in the last 3 months; (7) Participation in another clinical trial.

2.4.2.3 Subject Withdrawals

There will be at least one on-call clinician in each center. They would assess the severe adverse events (SAEs) and then determine whether the participant to continue or terminate the trial. Subjects may leave the study at their own discretion or the investigator may determine whether it is in the best interest of subjects to withdraw from the trial due to worsening of symptoms, or the occurrence of a serious adverse event.

2.4.2.4 Subject Recruitment, Screening and Group Assignment

Two strategies will be used to recruit participants with MRM. One is to recruit participants in outpatient clinics from the four centers mentioned above. The other is to show recruitment posters outside the clinics. Research assistants of each center will preliminarily screen the participants by recording their disease condition, history of the disease and treatment, and the demographic data. Experienced neurologist in each trial center will take charge of the diagnosis of MRM. Potential participants will receive a 3-cycle baseline assessment, during which they have to fill out headache diaries. Eligible participants then will be randomized to acupuncture or medication group. Acupuncturists will be in charge of participant assignment, and the trial procedures. Acupuncturists and neurologists will a be responsible for the assessment of safety during treatment, including the acupuncture-related

AEs or medication-related adverse events (AEs). During the trial, independent evaluators of each center will instruct the participants how to fill in their headache diaries. The evaluators will record the data on the case report form (CRF) through the whole trial period. The subject flow was shown in **Figure 1**.

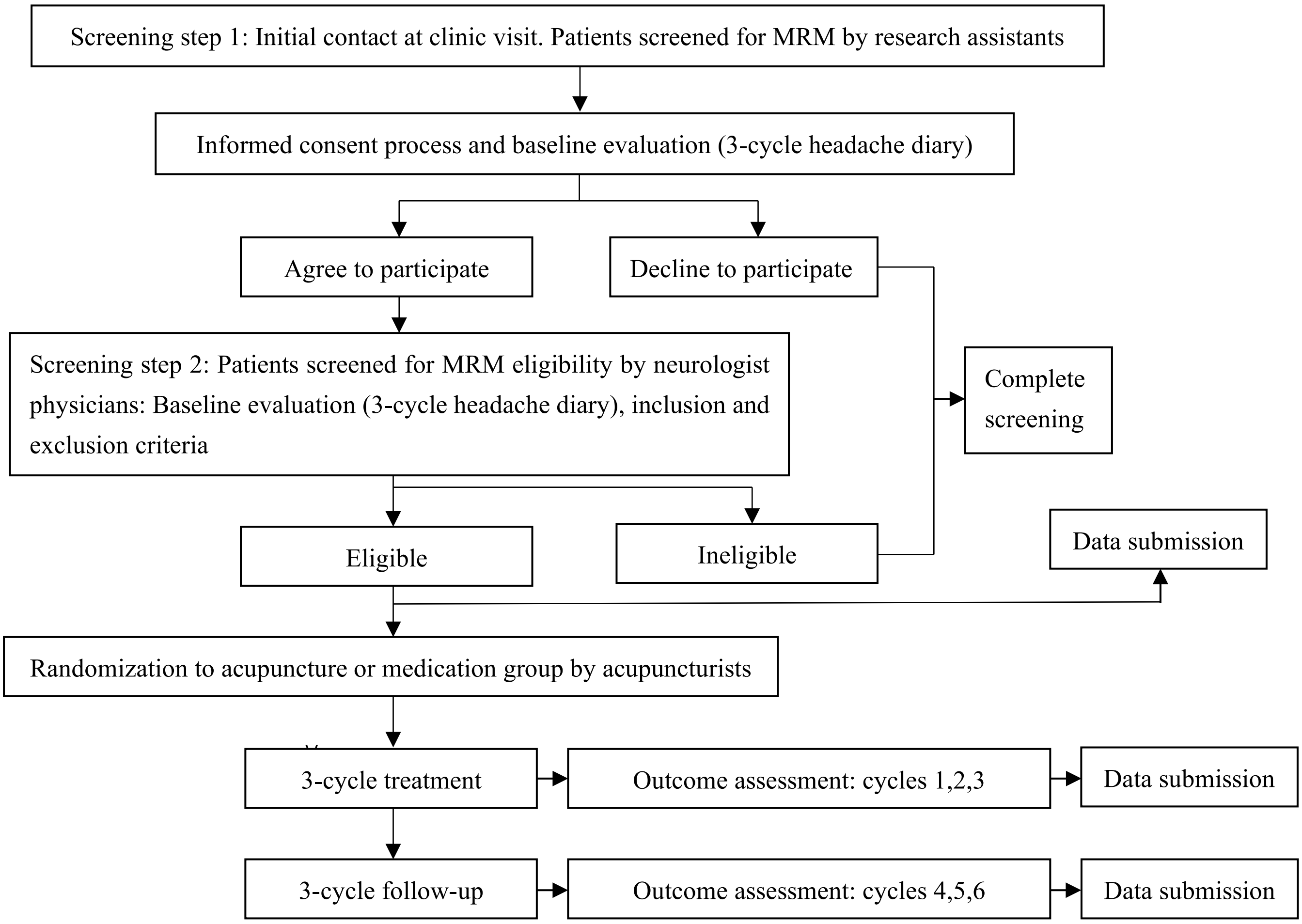

Figure 1. Subject flow

2.4.3 Trial flow Chart

The trial flow chart and the schedule of enrollment, interventions, and assessments were shown in **Figure 2** and **Figure 3**.

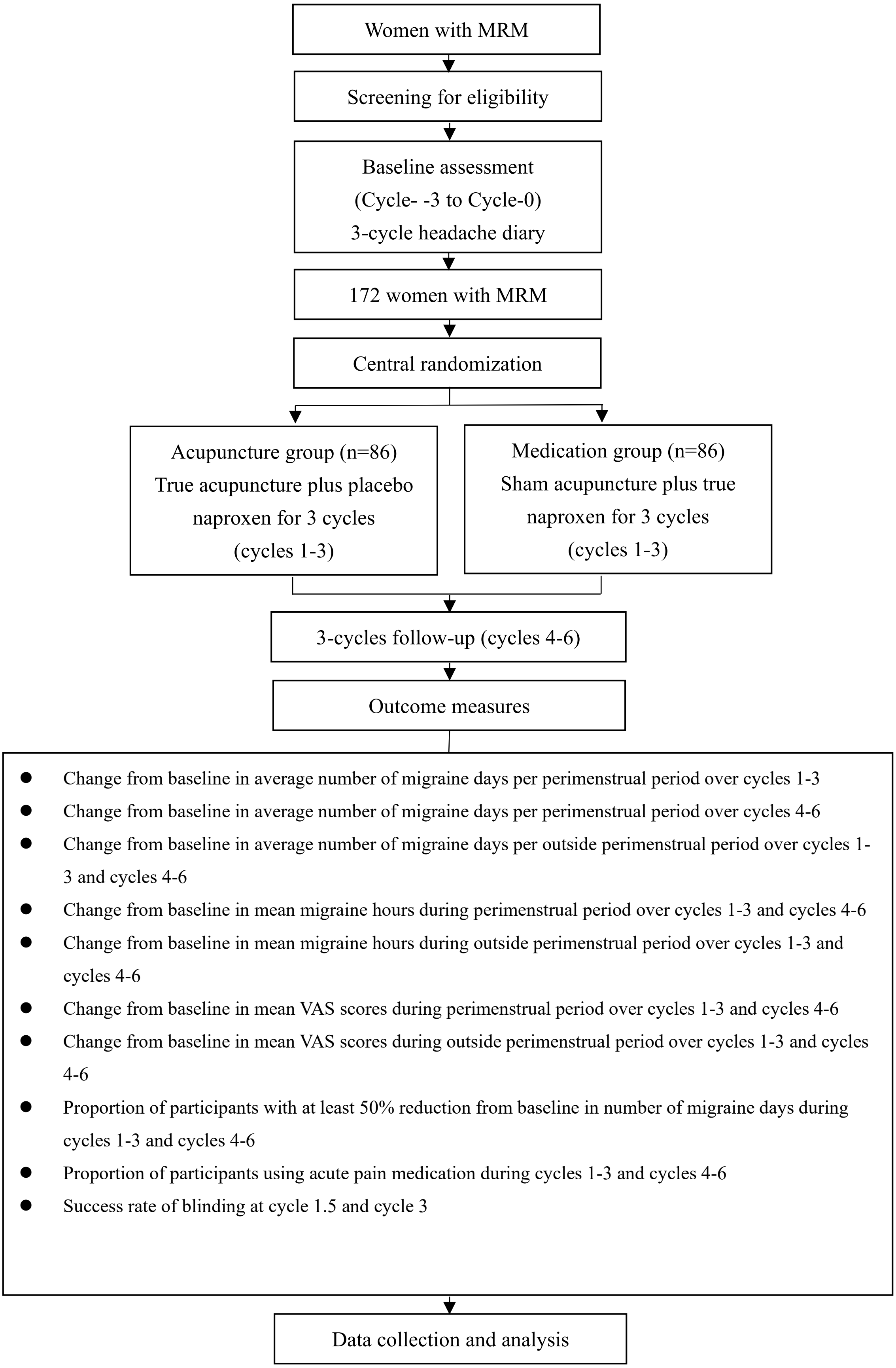

Figure 2. Trial flow chart

	STUDY PERIOD							
	**Baseline**	**Allocation**	**Treatment**			**Follow-up**		
**VISIT**	1	0	2	3	4	5	6	7
**TIMEPOINT(C, cycle)**	-3-0C		1C	2C	3C	4C	5C	6C
**Enrollment**								
Screening	×							
Informed consent	×							
Eligibility criteria	×							
Demography Characteristics	×							
Disease history of MRM	×							
Laboratory test	×				×			
Randomization		×						
**Interventions**								
True acupuncture plus placebo naproxen			×	×	×			
Sham acupuncture plus true naproxen			×	×	×			
**Assessments**								
Headache diary	×		×	×	×	×	×	×
Migraine days	×		×	×	×	×	×	×
Migraine hours	×		×	×	×	×	×	×
VAS	×		×	×	×	×	×	×
⩾50% migraine responder rate	×		×	×	×	×	×	×
Intake of acute pain medication	×		×	×	×	×	×	×
Assessment of blinding				×(1.5C)	×(3C)			
Participant's compliance			×	×	×			
Adverse events	×	×	×	×	×	×	×	×

MRM: menstrually related migraine; VAS: visual analogue scale.

Figure 3. The schedule of enrollment, interventions, and assessments

2.4.4 Outcomes Measurements

2.4.4.1 Primary Outcome

The primary outcome will be the change from baseline in average number of migraine days per perimenstrual period over cycles 1-3, assessed using headache diary data(14).
Calculation methods:
Average number of migraine days per perimenstrual period over cycles 1-3 is calculated as the cycle average, equals the total of migraine days during perimenstrual period over cycle 1, cycle 2 and cycle 3 divided by 3.

The frequency and severity of migraine will be based on data from headache diary recorded by participants on baseline (Cycle- -3 to Cycle-0), treatment phase (Cycle-1 to Cycle-3) and follow-up phase (Cycle-4 to Cycle-6). In the diaries, the participants recorded on diary cards with details of their migraine attacks, including the time of headache attack and cease, intensity, frequency, location (the forehead, top, temporal, and back of the head), cause of the headache, concomitant symptoms, and acute pain medications (if any) for each migraine attack. Additionally, participants were required to record information of their menstruation and the dates of receiving acupuncture treatments in their headache diaries. If acute pain medications were taken, participants were required to document the name, dosage, and time of taking the medicine, time of pain relief, and side effects of acute pain medication. In each center, data of headache diaries will be checked by two full-time research assistants first, and then entered into e-headache diary database by two independent data entry clerks. The audited data of e-headache diary will be finally carried forward into effectiveness data by statisticians.

2.4.4.2 Secondary Outcomes

①Change from baseline in average number of migraine days per perimenstrual period over cycles 4-6(14).
Calculation methods:
Average number of migraine days per perimenstrual period over cycles 4-6 is calculated as the cycle average, equals the total of migraine days during perimenstrual period over cycle 4, cycle

5 and cycle 6 divided by 3.

② Change from baseline in average number of migraine days per outside perimenstrual period over cycles 1-3 and cycles 4-6(14).

Calculation methods of the average number of migraine days during outside perimenstrual period at different timepoints:

a. Average number of migraine days per outside perimenstrual period over cycles 1-3 is calculated as the cycle average, equals the total of migraine days during outside perimenstrual period over cycle 1, cycle 2 and cycle 3 divided by 3;

b. Average number of migraine days per outside perimenstrual period over cycles 4-6 is calculated as the cycle average, equals the total of migraine days during outside perimenstrual period over cycle 4, cycle 5 and cycle 6 divided by 3.

③ Change from baseline in mean migraine hours during perimenstrual period over cycles 1-3 and cycles 4-6(14).

Calculation methods of the mean migraine hours during perimenstrual period at different timepoints:

a. Mean migraine hours during perimenstrual period over cycles 1-3 is calculated as daily average, equals the total of migraine hours during perimenstrual period divided by number of migraine attacks over cycles 1-3.

b. Mean migraine hours during perimenstrual period over cycles 4-6 is calculated as daily average, equals the total of migraine hours during perimenstrual period divided by number of migraine attacks over cycles 4-6.

④ Change from baseline in mean migraine hours during outside perimenstrual period over cycles 1-3 and cycles 4-6(14).

Calculation methods of the mean migraine hours during outside perimenstrual period at different timepoints:

a. Mean migraine hours during outside perimenstrual period over cycles 1-3 is calculated as daily average, equals the total of migraine hours during outside perimenstrual period divided by number of migraine attacks over cycles 1-3.

b. Mean migraine hours during outside perimenstrual period over cycles 4-6 is calculated as

daily average, equals the total of migraine hours during outside perimenstrual period divided by number of migraine attacks over cycles 4-6.

⑤ Change from baseline in mean pain visual analogue scale (VAS) during perimenstrual period over cycles 1-3 and cycles 4-6(16).

Calculation methods of the mean VAS scores during perimenstrual period at different timepoints:

a. Mean VAS during perimenstrual period over cycles 1-3 is calculated as daily average, equals the total of VAS score during perimenstrual period divided by number of migraine attacks over cycles 1-3.

b. Mean VAS during perimenstrual period over cycles 4-6 is calculated as daily average, equals the total of VAS score during perimenstrual period divided by number of migraine attacks over cycles 4-6.

VAS is widely accepted as one of the major outcomes for headache intensity. VAS-Pain scales are 0 to 10 where 0 = no pain, 10= pain as bad as can be.

⑥ Change from baseline in mean pain visual analogue scale (VAS) during outside perimenstrual period over cycles 1-3 and cycles 4-6(16).

Calculation methods of the mean VAS scores during outside perimenstrual period at different timepoints:

a. Mean VAS during outside perimenstrual period over cycles 1-3 is calculated as daily average, equals the total of VAS score during outside perimenstrual period divided by number of migraine attacks over cycles 1-3.

b. Mean VAS during outside perimenstrual period over cycles 4-6 is calculated as daily average, equals the total of VAS score during outside perimenstrual period divided by number of migraine attacks over cycles 4-6.

⑦ ≥50% migraine responder rate(14).

Calculation methods:

Proportion of participants with at least 50% reduction from baseline in number of migraine days during cycles 1-3 and cycles 4-6.

⑧ Intake of acute pain medication(14).

Calculation methods:

Proportion of participants using acute pain medication during cycles 1-3 and cycles 4-6.

⑨ Blinding Assessment

To confirm the blinding of participants, a questionnaire of blinding test will be conducted for all participants in the middle of treatment phase (Cycle-1.5) and at the end of treatment phase (Cycle-3). Participants in the four centers will be asked to guess which group they had been allocated.

3. Safety Assessment

All the serious adverse events (SAEs) and adverse events (AEs) will be recorded and measured by both participants themselves and clinicians including neurologists and acupuncturists through the whole trial. In our trial, the SAEs will be defined as events requiring hospitalization, causing disability or impaired ability to work, threatening life or resulting in death. AEs will be categorized as acupuncture related or medication related based on its potential association with acupuncture needling procedure by acupuncturists or medication intake procedure within 24 hours. The acupuncture related AEs defined as follows: broken needle, needle phobia, intense pricking, pricking lasting more than half an hour (no matter how intense it is) after acupuncture, hematoma, dermorrhagia, infection, abscess formation at the needling site, other discomfort induced by acupuncture (such as lassitude, numbness, somnolence, nausea, vomiting, palpitation, dizziness, headache, loss of appetite, insomnia, etc), and aggravation of existing symptoms, etc. Given that acupuncture is a minimally invasive therapy inevitably causing pain, pricking with a spontaneously remission within 30 min after acupuncture will be not regarded as acupuncture related AE. The medication related AEs defined as follows: nausea, palpitations, dyspepsia, upper abdominal pain, heartburn, somnolence, dizziness, and sweating attack, etc. The numbers of participants and events of AEs will be recorded. We will compare the proportion of participants who get treatment-emergent adverse events (TEAEs). Participants who get TEAEs at least once will be counted in for the comparison of the proportion.

4. Interventions

The intervention scheme of this trial will be based on expert consensus and result of

previous pilot study(23). There will be 1 full-time acupuncturists per center (4 acupuncturists in total in 4 centers) responsible for operation of acupuncture and sham acupuncture. In general, acupuncturists will be assigned to the same participants throughout the 3-cycle treatment schedule, except for vacation conflicts and staff turnover. All acupuncture treatments were delivered by acupuncturists who were registered with Ministry of Health of the People's Republic of China with more than 20 years of clinical experience. Sterile disposable steel acupuncture needle (size: 0.25 mm×25 mm for head points, 0.3 mm×40 mm for limb and abdomen points, Hwato Needles, made in Suzhou, China) will be used in this trial. It took 9 cycles in total for a patient to complete the trial, 3 cycle's baseline assessment, 3 cycle' treatment, and 3 cycle' follow up.

4.1 Treatment of the acupuncture group

In the acupuncture group, participants will be given true acupuncture plus placebo naproxen (2 tablets each time, once per day, will start three days before each predicted onset of the menstruation and continue during the end of their menstrual cycle for 3 cycles). In the event that menstruation begins later than predicted, no adjustment to the treatment will be made. If the MRM occurs earlier than predicted, participants will be asked to begin taking placebo naproxen immediately and one day earlier in the next cycle in an attempt to provide prophylactic coverage. The timing of treatment will be based on information collected (a headache diary) during the baseline phase and will not account for variability in menstruation and migraine onset during the treatment phase. The placebo naproxen will be identical in taste, smell and appearance as true naproxen sustained release tablets.

Acupuncture will consist of preventive treatment (two sessions each week) and premenstrual conditioning treatment (at least three sessions during 10 days before the predicted onset of each menstruation), deliver for 3 cycles. Each session will last for 30 minutes. The acupuncture points for preventive treatment will include both standard and additional points. The standard points are GV20 (Baihui), GV24 (Shenting), GB13 (Benshen), GB8 (Shuaigu), TE20 (Jiaosun), and GB20 (Fengchi). Additional points will be chosen individually depending on the syndrome differentiation of meridians in the headache region: TE5 (Waiguan) and GB34

(Yanglingquan) for Shaoyang headache; LI4 (Hegu) and ST44 (Neiting) for Yangming headache; BL60 (Kunlun) and SI3 (Houxi) for Taiyang headache; LR3 (Taichong) and GB40 (Qiuxu) for Jueyin headache; PC6 (Neiguan) for nausea and vomiting, and LR3 (Taichong) for dysphoria and susceptibility to rage. For premenstrual conditioning, each participant will have standard acupuncture points, KI12 (Dahe), CV3 (Zhongji) and ST29 (Guilai). All of the selected acupuncture points will be determined based on our previous research (16). After sterilizing the skin, the needle will be inserted 10 to 15mm in depth and manipulated by rotation methods to produce a characteristic sensation known as de qi (feeling of needle sensation refers to tenseness around the needle felt by the practitioner and numbness, distension, soreness, and heaviness around the point felt by the patient) at each point. Ten to twelve sterile disposable steel needles in each session will be used for acupuncture.

4.2 Treatment of medication group

In the medication group, participants will be given naproxen sustained release tablets (Diao Group Chengdu Pharmaceutical LTD, Chengdu, China) plus sham acupuncture. Naproxen sustained release tablets will be taken with a peri-menstrual dosing strategy (250mg/tablet, 2 tablets each time, once per day), which will start three days before each predicted onset of the menstruation and continue until the end of their menstrual cycle for 3 cycles. In the event that menstruation begins later than predicted, no adjustment to the treatment will be made. If the MRM occurs earlier than predicted, participants will be asked to begin taking naproxen immediately and one day earlier in the next cycle in an attempt to provide prophylactic coverage. The timing of treatment will be based on information collected (a headache diary) during the baseline phase and will not account for variability in menstruation and migraine onset during the treatment phase.

The method of applying blunt needles on effective acupuncture points will be not used in this trial as control for following reason: patients in China are very familiar with the sensations caused by acupuncture and therefore they may easily identify the blunt needles.

To make the needling stimulation consistent, the same type, size, and number of needles will be used for both groups. In order to avoid possible therapeutic effects of sham acupuncture,

the non-effective acupoints are chosen by the following three rules: (1) Acupuncture points that are defined as unrelated to headache or menstruation based on a vast amount of Chinese medicine reference books (26 ancient Chinese books of acupuncture, three Chinese acupuncture textbooks and >100 acupuncture research literatures); (2) 15 acupuncture points in the vicinity of elbow and knee joints are selected while the acupuncture points on the head, hands, feet, and trunk are excluded; (3) To mimic the nature of selecting points based on syndrome differentiation, the 15 sham points in the vicinity of elbow and knee joints are further randomly assigned to 3 subgroups: B, C, D. Each subgroup will have two points on the arms and three points on the legs. The participants in the medication group will be further randomly assigned into one of these three subgroups via central randomization system. Participants will be treated individually to avoid communication on the treatments they received.

4.3 Permitted and prohibited concomitant treatments

Throughout the whole trial, participants will be discouraged from any prophylactic medications of MRM, such as other non-steroidal anti-inflammatory drugs (NSAIDs), triptans, estrogen, magnesium, dihydroergotamine (DHE), methysergide and vitamin E. For any treatment already used, related information should be recorded in case report form.

5. Informed Consent

Informed Consent: Study Introduction

Dear women participants:

If your doctor thinks you have menstrually related migraine (MRM), we invite you to participate in this study aiming to evaluate the efficacy and safety of acupuncture in the preventive treatment of MRM. This study is supported and funded by the Beijing Foundation for Science and Technology of Traditional Chinese Medicine (JJ2011-03).

Before you decided to participate in the study, please read the following information carefully. It is helpful for you to know this study, understand why the study is performed, the study procedures, the duration and benefits of the study, risks and potential discomforts during and after study participation. If you like, you can also discuss this study with your

relatives and friends, or consult doctors for explanation and help to make the decision.

Introduction

I. Background and purposes

Menstrual migraine affects 20% of female migraineurs and most of such attacks are without aura. Menstrual migraine can be divided into two subtypes: pure menstrual migraine (PMM) and menstrually related migraine (MRM), with most of the cases being MRM. Pharmacotherapies, such as naproxen, triptans, estrogen, magnesium, and dihydroergotamine, are recommended for MRM prevention. However, these treatments are often associated with an increased risk of adverse events. Excessive use of pharmacotherapies may cause medication overuse–induced headache and an increase in headache frequency. Due to these limitations associated with the conventional treatments, efforts have been made to identify other effective as well as low-risk interventions for MRM. Acupuncture is one of the most commonly researched and widely accepted complementary and alternative medicine therapies for the treatment of migraine. In this study, a randomized controlled trial design will be used and we aim to evaluate the efficacy and safety of acupuncture in the preventive treatment of MRM. This study will be carried out simultaneously in 4 class A tertiary hospitals, and we expect a total number of 172 participants for voluntary participation.

II. Exclusion criteria

(1) chronic migraine, tension-type headache, cluster headache and other primary headaches;

(2) secondary headache and other neurological diseases;

(3) relatively severe systemic diseases (cardiovascular disease, acute infectious disease, hematopathy, endocrinopathy and allergy);

(4) headache caused by otorhinolaryngological diseases or intracranial pathological changes;

(5) oral contraceptives, pregnancy, or lactation period;

(6) use of prophylactic migraine medication in the last three months;

(7) involved in other clinical trials.

III. What to do next, if you decide to participate?

1. Before your enrollment in the study, you will receive the following exams to determine whether you are eligible to participate in the study:

The doctor will ask and record your medical history and perform related physical examination.

You will be required to complete 3-cycle headache diaries (baseline phase) for the confirmation of your diagnosis and inclusion criteria.

2. If the results of the above screening examinations meet the inclusion criteria and you are willing to participate in this study, you will be invited to continue study participation in the following steps:

(1) Based on the random number generated from the computer, the doctor will assign you to either the acupuncture or medication groups. Participants in the acupuncture group will receive acupuncture at traditional acupoints plus placebo naproxen; participants in the medication group will receive sham acupuncture at non-effective acupoints plus naproxen.

(2) In the study, Hwato brand disposable steel needles (Hwato Needles, made in Suzhou, China) will be used. Needle size: 0.25 mm×25 mm for head points, 0.3 mm×40 mm for limb and abdomen points. Frequency and duration of acupuncture treatment: 2 sessions each week for preventive treatment and at least three sessions during 10 days before the predicted onset of each menstruation for premenstrual conditioning treatment during cycles 1-3. The patients will receive 24 sessions of treatment in total.

(3) In the study, Naproxen sustained release tablets (Diao Group Chengdu Pharmaceutical LTD, Chengdu, China) will be used. Peri-menstrual dosing strategy: 500mg (250mg/table, 2 tablets) each time, once per day, started three days before each predicted onset of the menstruation and continue until the end of their menstrual cycle for 3 cycles. In the event that menstruation begins later than predicted, no adjustment to the treatment will be made. If the MRM occurs earlier than predicted, you will be asked to begin taking naproxen immediately and one day earlier in the next cycle in an attempt to provide prophylactic coverage. The timing of treatment will be based on information collected (a headache diary) during the baseline phase and will not account for variability in menstruation and migraine onset during the treatment phase.

(4) The duration of this study is 9 cycles, including 3-cycle baseline, a treatment period of 3 cycles, and a follow-up period of 3 cycles.

(5) During the study period, you need to record detailed headache diary faithfully. After treatment, you will need to hand in your diary to the doctor timely, and the doctor will record your signs and symptoms in detail.

3. Other requirements for your cooperation

As a participant of this study, you will have some relevant responsibilities, such as adherence to the schedule for examination, treatment, and outpatient follow-up. Additionally, you are also responsible for reporting any changes in your physical and mental status to your doctor during the study process regardless of whether you think these changes are related to the study or not.

You should follow the scheduled appointments with the doctor to come to the hospital for treatment (during follow-up, the doctor may get to know your conditions by phone or visiting your home). Your follow-up is very important because the doctor will determine whether the treatment that you are receiving really works, and the doctor will be able to guide the prevention and management of your symptoms timely.

During the study, you are not allowed to use other treatments for MRM. However, if acute pain medications are taken, please document the name, dosage, time of taking the medicine, time of pain relief, and side effects of acute pain medication.

IV. Potential benefits of study participation

You may benefit from this study. The benefits may include improvement of symptoms, even by medication group. The study may also help doctors and researchers to further evaluate the efficacy of acupuncture for MRM. The information will be beneficial in the management of other patients with a similar condition in the future. If you decide to participate in the study, you will get relevant physical and biochemical examination as well the study intervention for free during the study period.

V. Potential side effects, risks, discomforts, and inconveniences

The doctors will make every effort to prevent and treat any side effects brought on by this study. During treatment, you may feel soreness, numbness, heavy, distension sensation, etc.,

which are normal reactions to acupuncture. Acupuncture treatment may have some adverse effects, but it is rare and mild. You may feel fainting due to your individual physique or emotional stress when receive acupuncture needling. Your symptoms should be relieved after the cessation of acupuncture treatment and rest. Bleeding, hematoma, and other phenomena may occur after acupuncture treatment, and these phenomena should disappear after applying local pressure. If infection occurs in the needle site, your doctor will handle it timely. Medication treatment may also have some adverse effects as follows: nausea, palpitations, dyspepsia, upper abdominal pain, heartburn, somnolence, dizziness, and sweating attack, etc. With the treatment following the study protocol in the study, if you experience adverse reactions and events related to acupuncture or medication treatment, please feel free to call your doctor for help. The doctor will provide you timely treatment. If injuries have been confirmed and are caused by adverse reactions and events of the study, the study group will deal with them appropriately in accordance with relevant provisions. If you experience any discomfort or new change of your symptoms, or any other unforeseen circumstances during study period, regardless of whether these events is relevant with treatment of the study or not, you shall promptly notify your doctor, and he/she will evaluate the condition and give you appropriate medical treatment.

VI. Payments/compensation for participation

If you participate in the study, during the study, you will get relevant physical, biochemical examination, acupuncture and medication treatment for free. If adverse events occur during the study, they will be managed accordingly by medical experts who will also identify whether they are related to the study or not. The treatment and examination required for your concomitant diseases non-related to the study will not be free of charge.

VII. Confidentiality of personal information

All the information related to your participation in this study will be kept confidential by the institute where your participation takes place. Only the institutes responsible for the study, clinical research institutes, and ethics committees may have access to your medical records. Your name will not appear in any publication or report related to this study. We will make every effort to protect the privacy of your personal medical information as per legal requirements and

laws.

VIII. How to acquire extra information?

You can ask any questions about the study at any time and will get answers timely. If we notice any new information that may affect your willingness and decision to continue participating in the study, the doctor will keep you informed.

IX. Can you voluntarily choose to participate in or withdraw from the study?

Whether to participate in this study or not entirely depends on your desire. You can refuse to participate in the study, or withdraw from the study at any time during the study, which will not affect the relationship between you and your doctor and will not affect your medical interests or interests in other areas. For the consideration of your best interests, doctors or researchers may terminate your participation in this study at any time. If you withdraw from the study for any reason, you may be asked for information related of acupuncture and medication treatment or the use of other medications during your participation of the study. If the doctor considers it necessary, you may also be asked to have some laboratory tests and physical examinations performed.

X. What you need to do now?

Decide whether to participate in this study or not. Before you make the decision to participate in the study, please ask your doctor if you have any concerns.

Thank you for reading the above information. If you decide to participate in this study, please tell your doctor, he/she will help you make arrangement for the study.

Please keep this document for your own record.

Informed Consent: Signature Page

Study title: Acupuncture for menstrually related migraine prophylaxis: a single-blinded, double-dummy, multicenter randomized controlled trial

Organizer of this study: Beijing Hospital of Traditional Chinese Medicine, Capital Medical University

Statement of agreement:

I have read the above information about this study and have the opportunity to discuss this study with my doctor and ask questions. All my questions were answered satisfactorily. I

understand the potential risks and benefits from participation in this study. I understand the participation of the study is voluntary and I confirm that I was given sufficient time for consideration of study participation. I confirm that I understand that:

I can always ask the doctor for additional/more information.

I can withdraw from the study at any time without discrimination or retaliation and my medical treatment and interests will not be affected.

I understand that if I withdraw from the study, I will tell the doctor the changes of my disease condition and complete the relevant physical and biochemical examinations if needed, which will be very helpful for the whole study.

If I need to take any other medications due to the changes of my medical condition, I will seek medical advice from the doctor beforehand or afterwards tell the doctor truthfully.

I agree to allow the research institute, collaborative institutes, and ethics committees to inspect the data relevant to my study participation.

I will receive a signed and dated copy of the informed consent form.

Finally, I decide and agree to participate in this study and ensure the adherence to doctor's orders to the best I can.

Signature of patient: Year month day

Telephone:

I confirm that I have explained this study in detail to the patient, including patient's rights as well as the potential benefits and risks, and have given the patient a signed copy of the informed consent form.

Signature of doctor: Year month day

Office phone number of doctor:

6. Quality Control

To guarantee the quality of the study, the trial protocol will be reviewed and may be revised by expert acupuncturists, neurologists, and statisticians several times. A central randomization system will be adopted to avoid selection bias. Strict eligible criteria will be pre-set to restrict the research population. Blinded effect assessment and blinded statistics will be designed to

guarantee the objectivity of the data. All research staffs, especially the research assistants, acupuncturists and data entry clerks, will be required to attend a series of training on how to use the central randomization system and data entry system, how to fill the case report form and headache diary, how to manipulate interventions correctly, and how to assess the outcomes, etc. A double-entry method will be used in this trial. The data of therapeutic evaluating will be calculated by the statisticians. A three-level inspection plan will be designed for quality inspecting.

7. Data Management

7.1 The Raw Data Management and Archiving

We will use Remote Data Capture (RDC) system to perform data entry. The research assistants will fill out all the electrical CRF through RDC system. Researchers will inspect the eCRF, and signed electrically for the eCRF going into effect. The eCRF and the trace of eCRF revising will be left in the Oracle database.

7.2 Data Entry and Storage

7.2.1 Database Building and Testing, Data Entry Interface

The eCRF will be noted through CDISC CDASH standard, and the data entry interface will be generated through the Oracle Clinical software. The data entry interface should be in accordance with the paper version CRF as far as possible. The inputted data will be stored in the Oracle database. After preliminarily setting up the database, the entry clerks will input some analog data according to the CRF to test the database. The testing contains: (1) the agreement of the data entry interface and the paper version CRF; (2) the agreement of the exported data from the database and the analog data. After the testing, data administrators should revise the database and make a testing report. Then they electrically signed on the approval page of the database to indicate that the testing is completed. The electrical files of the analog CRF, Noted CRF, screenshot of the data entry interface, database testing report, and the approval page of the database should be saved. If the database updates during the trial, the electrical files

mentioned above are also need to be updated.

7.2.2 Data Entry and Inspection

The research assistants take charge of the data entry for our trial. Before the entry, all the research assistants will accept the related training according to the data entry handbook. Researchers will inspect the database, and then sign electrically to let the data go in to effect.

7.3 Data Verification and Problems Solving

Researchers will verify the data through Data Verification Plan (DVP) approved by the data administrator and the statisticians. Data queries will be inputted to a data query database, and form the Data Clarification Form (DCF). After being inspected, the DCF will then be handed back to the original center, and the researchers of the center should answer the queries. Any revision of the database will be recorded through the RDC software.

7.4 Medical Coding

A data administrator who has the medicine background will take charge of the medical coding. The contents of the coding are the clinical history, adverse events, and combined medication. The clinical history and adverse events will be coded through MedDRA dictionary (Version 13.0), and the combined medication will be coded via WHO DD dictionary (Version 2007.03). The lead researchers will verify the coded e-files.

7.5 Data Blinding Review and Data Management Report

When the data checking is finished, a data auditing and blinding review meeting will be hold. On the meeting, the data administrators, statisticians, researchers, clinical inspectors, and other related members would have a discussion on the following items according to the data management report and the data lists:

•Distribution of the participants;

•Protocol deviation;

•Possible outlier;

•Baseline data review;

•Outcomes review;

•AEs review;

•Classifying plan of the statistical analysis population;

•Statistical analysis plan.

Participants will be classified to their suitable statistical analysis sets according to the definition in the protocol. No patient can be excluded from the analysis, unless getting the permission of the meeting participants. All the meeting participants should sign the data locking consent, and the data auditing resolution.

7.6 Database Locking

The database will be locked if it fulfills all the aspects as followed: All the queries have been solved, and the database has been updated; No query has been found through the data inspection; The medical coding has been completed; The plan of the participants' classification has been approved; The final draft of the SAP has been made, and approved by the project leader.

The statisticians and the data administrators will sign the data locking form, and then the database will be locked. The locked database will be sent to the statisticians for further statistical analysis through the data format of SAS.

8. Statistical consideration

The following is an overview of the statistical considerations. Details of the pre-specified statistical analyses can be found in the Statistical Analysis Plan (SAP).

8.1 Statistical Analysis

The primary study hypothesis is that acupuncture is different from medication in decreasing migraine days for women with MRM. The primary outcome is the change from baseline in average number of migraine days per perimenstrual period over cycles 1-3, assessed using headache diary data. The primary analysis will be intention-to-treat with the last observation carried forward (LOCF) method. Migraine days during perimenstrual period will be

summarized in each treatment group and compared using mixed-effect model.

The following secondary outcomes will be analyzed using the mixed-effect model, *t* test, Wilcoxon rank-sum test, Chi-square test or Fisher's exact test, as appropriate and the intent-to-treat principal:

1. Change from baseline in average number of migraine days per perimenstrual period over cycles 4-6
2. Change from baseline in average number of migraine days per outside perimenstrual period over cycles 1-3 and cycles 4-6
3. Change from baseline in mean migraine hours during perimenstrual period over cycles 1-3 and cycles 4-6
4. Change from baseline in mean migraine hours during outside perimenstrual period over cycles 1-3 and cycles 4-6
5. Change from baseline in mean pain VAS during perimenstrual period over cycles 1-3 and cycles 4-6
6. Change from baseline in mean pain VAS during outside perimenstrual period over cycles 1-3 and cycles 4-6
7. Proportion of participants with at least 50% reduction from baseline in number of migraine days during cycles 1-3 and cycles 4-6
8. Proportion of participants using acute pain medication during cycles 1-3 and cycles 4-6
9. Success rate of blinding at cycle 1.5 and cycle 3

A two-side test with $p<0.05$ will be considered significant for all analyses.

8.2 Statistical Analysis Plan (SAP)

Prior to database lock and before code breaking, a final version of the SAP shall be issued and approved by the study statistician, and the principal investigator. The SAP will define all 'pre-specified, planned analyses' and provide the general specifications for the analysis of the data to be collected and presented in the Clinical Study Report.

9. Ethical principle

For every study center, only when the trial protocol is approved by the Investigational Review Board (IRB), the enrollment of participant will begin, but all should be after April 27, 2013.

10. Funding

This study is supported and funded by the Beijing Foundation for Science and Technology of Traditional Chinese Medicine (JJ2011-03).

11. Update on the Published Protocol

As compared to the published protocol (Zhang XZ, Zhang L, Guo J, et al. Acupuncture as prophylaxis for menstrual-related migraine: study protocol for a multicenter randomized controlled trial. Trials. 2013;14:374), the present finalized study protocol had made several amendments because of practicality.

(1) langue revision of outcomes for precision presentation and readers' better understanding: a, 'The change of migraine days inside the menstrual cycle' was revised to 'The change from baseline in average number of migraine days per perimenstrual period'; b, 'The change of migraine days outside the menstrual cycle' was revised to 'The change from baseline in average number of migraine days per outside perimenstrual period '; c, 'Duration of migraine attack' was revised to 'The change from baseline in mean migraine hours during/outside perimenstrual period'; d, 'Visual analogue scale (VAS) for pain' was revised to 'The change from baseline in mean pain VAS during/outside perimenstrual period'; e, 'The proportion of responders (defined as the proportion of patients with at least a 50% reduction of the number of menstrual migraine days)' was revised to 'The proportion of participants with at least 50% reduction from baseline in number of migraine days'; f, ' Intake of acute medication' was revised to 'The proportion of participants using acute pain medication'.

(2) Major amendments for mistake correction or better presentation (Table 1):

Table 1. Major update of the published protocol

No.	Item	Published version	Final version
1	Study design	Follow-up phase: 1 month.	Follow-up phase: 3 cycles (Cycle-4 to Cycle-6).
2	Sample size	According to the previous pilot study, the number of migraine days after treatment in acupuncture group is 3.1 ± 2.7 days, and the control group is 5.2 ± 4.4 days. Based on 0.8 power to detect a significant difference(α = 0.01, two-sided), 73 participants will be required for each group, which is calculated by PASS 2008. Allowing for a 20% withdrawal rate, we will plan to enroll a total of 184 participants with 92 participants in each group.	According to the previous pilot study, the number of migraine days after treatment in acupuncture group is 3.1 ± 2.7 days, and the control group is 5.2 ± 4.4 days. Based on 0.9 power to detect a significant difference(α = 0.05, two-sided), 68 participants will be required for each group, which is calculated by PASS 2008. Allowing for a 20% withdrawal rate, we will plan to enroll a total of 172 participants with 86 participants in each group.
3	Randomization	The central randomization will be performed by the Research Center of Clinical Epidemiology affiliated to Peking University in China, which use block randomization to generate the random allocation sequence and prepare predetermined computer made randomization opaque sealed envelopes.	We randomly assigned each participant to acupuncture or medication groups via a central randomization system using a 1:1 ratio, which was performed by the Research Center of Clinical Epidemiology affiliated to Peking University. Randomization was stratified by centers with a fixed block size of 4.
4	Primary outcome	a. The change of migraine days inside the menstrual cycle<br>b. The proportion of responders (defined as the proportion of patients with at least a 50% reduction of the number of menstrual migraine days).	Change from baseline in average number of migraine days per perimenstrual period over cycles 1-3.

References

1. Stewart WF, Shechter A, Rasmussen BK. Migraine prevalence. A review of population-based studies. Neurology. 1994;44(6 Suppl 4):S17-23.
2. Lipton RB, Stewart WF, Diamond S, Diamond ML, Reed M. Prevalence and burden of migraine in the United States: data from the American Migraine Study II. Headache. 2001;41(7):646-57.
3. Granella F, Sances G, Allais G, Nappi RE, Tirelli A, Benedetto C, et al. Characteristics of menstrual and nonmenstrual attacks in women with menstrually related migraine referred to headache centres. Cephalalgia. 2004;24(9):707-16.
4. Stewart WF, Lipton RB, Celentano DD, Reed ML. Prevalence of migraine headache in the United States. Relation to age, income, race, and other sociodemographic factors. JAMA. 1992;267(1):64-9.
5. Pryse-Phillips W, Findlay H, Tugwell P, Edmeads J, Murray TJ, Nelson RF. A Canadian population survey on the clinical, epidemiologic and societal impact of migraine and tension-type headache. Can J Neurol Sci. 1992;19(3):333-9.

6. Epstein MT, Hockaday JM, Hockaday TD. Migraine and reporoductive hormones throughout the menstrual cycle. Lancet. 1975;1(7906):543-8.
7. Newman LC, Lipton RB, Lay CL, Solomon S. A pilot study of oral sumatriptan as intermittent prophylaxis of menstruation-related migraine. Neurology. 1998;51(1):307-9.
8. Nattero G. Menstrual headache. Adv Neurol. 1982;33:215-26.
9. Couturier EG, Bomhof MA, Neven AK, van Duijn NP. Menstrual migraine in a representative Dutch population sample: prevalence, disability and treatment. Cephalalgia. 2003;23(4):302-8.
10. MacGregor EA, Hackshaw A. Prevalence of migraine on each day of the natural menstrual cycle. Neurology. 2004;63(2):351-3.
11. Lay CL, Payne R. Recognition and treatment of menstrual migraine. Neurologist. 2007;13(4):197-204.
12. Silberstein SD, Hutchinson SL. Diagnosis and treatment of the menstrual migraine patient. Headache. 2008;48 Suppl 3:S115-23.
13. MacGregor EA. Prevention and treatment of menstrual migraine. Drugs. 2010;70(14):1799-818.
14. Diener HC, Kronfeld K, Boewing G, Lungenhausen M, Maier C, Molsberger A, et al. Efficacy of acupuncture for the prophylaxis of migraine: a multicentre randomised controlled clinical trial. Lancet Neurol. 2006;5(4):310-6.
15. Yang CP, Chang MH, Liu PE, Li TC, Hsieh CL, Hwang KL, et al. Acupuncture versus topiramate in chronic migraine prophylaxis: a randomized clinical trial. Cephalalgia. 2011;31(15):1510-21.
16. Wang LP, Zhang XZ, Guo J, Liu HL, Zhang Y, Liu CZ, et al. Efficacy of acupuncture for migraine prophylaxis: a single-blinded, double-dummy, randomized controlled trial. Pain. 2011;152(8):1864-71.
17. Da Silva AN. Acupuncture for migraine prevention. Headache. 2015;55(3):470-3.
18. Endres HG, Diener HC, Molsberger A. Role of acupuncture in the treatment of migraine. Expert Rev Neurother. 2007;7(9):1121-34.
19. Melzack R, Wall PD. Pain mechanisms: a new theory. Science. 1965;150(3699):971-9.
20. Carlsson C. Acupuncture mechanisms for clinically relevant long-term effects--reconsideration and a hypothesis. Acupunct Med. 2002;20(2-3):82-99.
21. Pielsticker A, Haag G, Zaudig M, Lautenbacher S. Impairment of pain inhibition in chronic tension-type headache. Pain. 2005;118(1-2):215-23.
22. Rossi P, Serrao M, Perrotta A, Pierelli F, Sandrini G, Nappi G. Neurophysiological approach to central pain modulation in primary headaches. J Headache Pain. 2005;6(4):191-4.
23. Li C, Liu H, Yang C. Clinical observation of acupuncture as prophylaxis for menstrually related migraine (MRM). Beijing Journal of Traditional Chinese Medicine. 2011;30:617-8.
24. Headache Classification Subcommittee of the International Headache S. The International Classification of Headache Disorders: 2nd edition. Cephalalgia. 2004;24 Suppl 1:9-160.

Acupuncture as prophylaxis for menstrually related migraine : A Multicenter, Randomized Controlled Trial

Statistical Analysis Plan

Final version: April 27, 2014

Trial Registration: ISRCTN registry 57133712

Prepared for and approved by the Executive committee of the study:

Dr. Linpeng Wang (Chair), Beijing Hospital of Traditional Chinese Medicine, Capital Medical University

Dr. Huilin Liu (Member), Beijing Hospital of Traditional Chinese Medicine, Capital Medical University

Prepared by an Independent Statistician:

Dr. Lin Zeng (Statistician), Research Centre of Clinical Epidemiology, Peking University Third Hospital

Table of Contents

1. Introduction 36
2. Study Objective 37
3. Design........ 37
    3.1 Overview........ 37
    3.2 Inclusion/Exclusion Criteria 37
        3.2.1 Inclusion Criteria........ 37
        3.2.2 Exclusion Criteria........ 37
4. Study Schema 39
5. Efficacy and Safety outcomes 41
    5.1 Efficacy outcomes........ 41
        5.1.1 Primary Efficacy outcome........ 41
        5.1.2 Secondary Efficacy outcomes 41
6. Statistical Considerations 41
    6.1 Study hypothesis 41
    6.2 Study Populations 42
    6.3 Statistical Analyses 42
        6.3.1 The general principle........ 42
        6.3.2 Demographics and Baseline Characteristics 44
        6.3.3 Analyses for Primary Outcome 44
        6.3.4 Analyses for Secondary Outcome 45
        6.3.5 Safety Analyses 45
    6.4 Update on the statistical analysis plan 46

1. Introduction

Menstrual migraine can be divided into two subtypes: pure menstrual migraine (PMM) and menstrually related migraine (MRM), with most of the cases being MRM. MRM is defined as attacks of migraine without aura, occurring between days -2 to +3 of the menstruation in at least two out of three menstrual cycles, with additional attacks of migraine at other times of the cycles by the International Classification of Headache Disorders (ICHD). More than 50% of female patients report that their migraines are associated with the menses. Attack onset is usually before the age of 20 years, with peak prevalence between the ages of 25 and 55 years, declining with menopause. Increasing evidence links menstrual migraine to the female sex hormones. Most of the data in the literature report that MRM causes significant limitations of daily activities (for example, nausea, vomiting, and photo-phonophobia), and the attacks are generally longer, more severe, and less drug-responsive than non-menstrual ones. The options available for the treatment of MRM include acute therapy and prophylaxis (short-term preventive therapy and long-term preventive therapy). Acute therapy is initiated first. As MRM is unique in its predictability, treatment can be targeted to the period of time when patients are most likely to experience migraine. The treatment window for menstrual migraine occurs between 2 days before and 3 days after the onset of menses. The goals of prophylactic strategies are to reduce attack frequency, severity and duration, improve responsiveness of treatment for acute attacks, improve function, and reduce disability. Prophylactic medications include non-steroidal anti-inflammatory drugs, triptans, estrogen, magnesium, dihydroergotamine, methysergide, and vitamin E. Caution should be taken when the drug is used within the same month for treating migraine attacks occurring during perimenstrual period and outside perimenstrual period due to the risk of medication-overuse headache, a risk which is, however, common to all medications used for menstrual-migraine prophylaxis or treatment. Several studies have already reported the encouraging results in the therapy for migraine by acupuncture. When reviewing the evidence of acupuncture for MRM, only one published randomized controlled trial could be found. However, because of the limited evidence, high-quality RCTs are needed to assess the efficacy of acupuncture as the preventive treatment for

MRM.

2. Study Objective

The primary objective is to evaluate the efficacy of acupuncture as the preventive treatment for menstrually related migraine.

3. Design

3.1 Overview

This multicenter, participant-blinded, randomized controlled clinical trial will be performed to demonstrate the safety and efficacy of acupuncture as the preventive treatment for MRM.

3.2 Inclusion/Exclusion Criteria

3.2.1 Inclusion Criteria

1.Diagnosis of MRM: MRM without aura (code A1.1.2), in which migraine without aura always occurs on days 1 ± 2 of menstruation in at least two out of three menstrual cycles, and at other times due to different triggering factors or for no apparent specific reason (International Classification of Headache Disorders-second edition [ICHD-II]);
2.Regular menstrual cycle(25-35 days);
3. Could predict within three days both the onset of menstruation and perimenstrual migraine attacks;
4. Repeated migraine attacks, frequency of non-menstrual migraine is more than once a month;
5. Written informed consent.

3.2.2 Exclusion Criteria

1. Chronic migraine, tension-type headache, cluster headache, and other primary headaches;

2. Secondary headache and other neurological diseases;

3. Relatively severe systemic diseases (cardiovascular disease, acute infectious disease, hematopathy, endocrinopathy, and allergy);

4. Headache caused by otorhinolaryngology diseases or intracranial pathological changes;

5. Oral contraceptives, pregnancy, or lactation period;

6. Use of prophylactic migraine medication in the last 3 months;

7. Participation in another clinical trial.

4. Study Schema

	STUDY PERIOD							
	**Baseline**	**Allocation**	**Treatment**			**Follow-up**		
**VISIT**	1	0	2	3	4	5	6	7
**TIMEPOINT(C, cycle)**	-3-0C		1C	2C	3C	4C	5C	6C
**Enrollment**								
Screening	×							
Informed consent	×							
Eligibility criteria	×							
Demography Characteristics	×							
Disease history of MRM	×							
Laboratory test	×				×			
Randomization		×						
**Interventions**								
True acupuncture plus placebo naproxen			×	×	×			
Sham acupuncture plus true naproxen			×	×	×			
**Assessments**								
Headache diary	×		×	×	×	×	×	×
Migraine days	×		×	×	×	×	×	×
Migraine hours	×		×	×	×	×	×	×
VAS	×		×	×	×	×	×	×
≥50% migraine responder rate	×		×	×	×	×	×	×
Intake of acute pain medication	×		×	×	×	×	×	×

Assessment of blinding				×(1.5C)	×(3C)			
Participant’s compliance			×	×	×			
Adverse events	×	×	×	×	×	×	×	×

MRM: menstrually related migraine; VAS: visual analogue scale.

Figure 1. The schedule of enrollment, interventions, and assessments

5. Efficacy and Safety outcomes

5.1 Efficacy outcomes

5.1.1 Primary Efficacy outcome

The primary efficacy endpoint will be the change from baseline in average number of migraine days per perimenstrual period over cycles 1-3, assessed using headache diary data.

5.1.2 Secondary Efficacy outcomes

1. Change from baseline in average number of migraine days per perimenstrual period over cycles 4-6
2. Change from baseline in average number of migraine days per outside perimenstrual period over cycles 1-3 and cycles 4-6
3. Change from baseline in mean migraine hours during perimenstrual period over cycles 1-3 and cycles 4-6
4. Change from baseline in mean migraine hours during outside perimenstrual period over cycles 1-3 and cycles 4-6
5. Change from baseline in mean pain visual analogue scale (VAS) during perimenstrual period over cycles 1-3 and cycles 4-6
6. Change from baseline in mean pain visual analogue scale (VAS) during outside perimenstrual period over cycles 1-3 and cycles 4-6
7. Proportion of participants with at least 50% reduction from baseline in number of migraine days during cycles 1-3 and cycles 4-6
8. Proportion of participants using acute pain medication during cycles 1-3 and cycles 4-6
9. Success rate of blinding at cycle 1.5 and cycle 3

6. Statistical Considerations

6.1 Study hypothesis

The primary study hypothesis is that acupuncture is different from medication in reducing

migraine days in women with menstrually related migraine.

6.2 Study Populations

All patients with randomization will be included in the analysis set regardless of whether they receive any treatment. According to the intention-to-treat principle, all analysis will be based on the randomization set. The intention-to-treat (ITT) set will include all randomized participants who have at least one treatment and one primary outcome measure. The per-protocol (PP) set will include all randomized participants who have no major protocol deviation. The safety set will include all randomized participants who receive at least one session of acupuncture.

6.3 Statistical Analyses

6.3.1 The general principle

Summary Statistics

Summary tables (descriptive statistics and/or frequency tables) will be provided for all variables at different endpoints. For continuous variables, means and standard deviations will be presented, unless the variable has a skewed distribution, in which case medians, 25th and 75th percentiles will be presented. For categorical variables, the number and percentage of participants within each category will be presented. For each variable (continuous or categorical), the number of missing values will be reported.

Statistical Comparisons Between Groups

Continuous variables will be compared using a *t* test or Wilcoxon rank-sum test if data show serious deviations from a normal distribution. Categorical data or ordinal data will be compared using a Chi-square test or Fisher's exact test, as appropriate. All tests will be two-sided.

For the analysis of the primary and secondary outcomes, estimated treatment differences and associated 95% two-sided confidence intervals will be presented.

Multicenter study

To estimate the overall variability of the center effects, we used the random center effects (RCE) accounting for center effects (1). Therefore, mixed-effect model was used for the primary outcome.

Missing data

Efficacy analyses will be conducted in the intention-to treat (ITT) set, which will include all randomized subjects of whom the migraine days are measured in at least on post-baseline 1 cycle.

Missing data will be imputed by the last observation carried forward (LOCF) method (2). In addition, to examine sensitivity of the LOCF method imputed the missing data, we will perform a sensitivity analysis with multiple imputation method under the missing at random (MAR) assumption (3) for the primary outcome with missing data. Multiple imputations use the observed data to fill in the missing values repeatedly to give rise to multiple "pseudo-complete" datasets. We will impute the missing data 100 times using the following one of methods 1) regression imputation, if data sets with monotone missing patterns, or 2) Markov chain Monte Carlo imputation, if data sets with other patterns. For this we will use the SAS procedure Proc MI process. Each method will give rise to 100 different imputed data sets. We will fit our final model described before to each of these imputed datasets and then compute an overall estimate of the intervention effect as an average of the imputation specific estimates. The standard error of the overall intervention effect estimate will be calculated using Rubin's formula. SAS procedure Proc MIANALYZE will be used to implement these tasks.

Multiple Comparisons

Since only one primary outcome is defined, no adjustments to the significance level will be required to account for multiple testing.

For the analysis of the secondary and safety outcomes, no adjustment for multiple comparisons will be made.

Analysis Software

For all statistical analyses, SAS 9.4 software will be used. All hypothesis testing will be carried out at the 5% (2-sided) significance level.

6.3.2 Demographics and Baseline Characteristics

All data recorded at baseline will be summarized by group. Comparisons between groups will be done using the methodology described in section 6.3.1. Summaries will be presented for the ITT Set in both groups.

6.3.3 Analyses for Primary Outcome

The cycle migraine days will be summarized by the mean and standard deviation (or median and interquartile range if data are skewed) in each group and compared using mixed-effect model adjusted for the baseline value, with treatment as a fixed effect, and center as a random effect. In case of serious violations of the model assumptions (normality and constant variance of the residuals), a log-transformation may be applied. If not appropriate, a Wilcoxon rank-sum test will be used. The effect of the treatment will be estimated by the difference (or ratio, in case of log-transformation) between treatments and will be presented along with its associated 95% confidence interval.

Sensitivity Analyses for the Primary Outcome

For the primary outcome, sensitivity analyses will be performed on the per-protocol (PP) set to assess the robustness of the study conclusion to the choice of analysis population. The PP set is a subset of the ITT, which includes all randomized participants who have no major protocol deviations.

In addition, sensitivity analyses will be performed using multiple imputation method for missing data to assess whether the primary outcome is robust to departure from imputed missing data using LOCF method.

There will be between and within-woman differences in menstrual cycle lengths which

inherently confound the outcomes. To evaluate this potential confounder, we will perform a sensitivity analysis that using menstrual cycle length as a control variable in a mixed-effect model.

6.3.4 Analyses for Secondary Outcome

Efficacy analyses for all secondary outcomes will be performed in the ITT population observed cases, with imputation of missing data using LOCF method.

The mixed-effect model (using baseline value as a covariate, treatment as a fixed effect, and center as a random effect) will also be used for secondary outcomes of the changes from baseline in average number of migraine days per outside perimenstrual period, mean migraine hours during perimenstrual period and outside perimenstrual period, mean pain visual analogue scale (VAS) during perimenstrual period and outside perimenstrual period. For proportion of participants with at least 50% reduction from baseline in number of migraine days and proportion of participants using acute pain medication will be compared between groups using a Chi-square test or Fisher's exact test, as appropriate.

6.3.5 Safety Analyses

Adverse events

All adverse events and serious adverse events will be listed. Adverse events include the acupuncture-related adverse events and medication-related adverse events. Chi-square test or Fisher's exact test will be used to compare the incidence of adverse events between the acupuncture group and medication groups.

Participant's compliance

The number and percentage of subjects who received at least 80% of the planned acupuncture treatment or medication will be analyzed with Chi-square test or Fisher's exact test.

Blinding assessment

The number and percentage of participants who guess 'true acupuncture plus placebo naproxen' when asked if they received true acupuncture plus placebo naproxen or sham acupuncture plus naproxen treatment will be summarized by treatment, and Kappa analysis will be used to determine whether participants correctly guess their group assignment at a higher rate than would be expected by chance.

6.4 Update on the statistical analysis plan

As compared to the initial statistical analysis plan (SAP) published in Trials (Zhang XZ, Zhang L, Guo J, et al. Acupuncture as prophylaxis for menstrual-related migraine: study protocol for a multicenter randomized controlled trial. Trials. 2013;14:374), The original Analysis Plan has been amended as follows:

Table 1. Major update of the statistical analysis plan

No.	Item	Published version	Final version
1	Primary outcome analysis	Analysis of variance (ANOVA) for repeated measures is used to compare the two groups. Comparisons within groups for the outcomes in each one of the time point are done using the Tukey's post hoc test of Multivariate Analysis of Variance (MANOVA). Meanwhile, stratified analysis within four different centers will be performed to control confounding factor if necessary.	Mixed-effect model for primary analysis using baseline value as a covariate, treatment as a fixed effect, and center as a random effect.
2			More details were provided for the sensitivity analyses of primary outcome in primary analysis (see 6.3.3).
3	Analysis Software	Every analysis is conducted by using the SPSS software version 13.0.	For all statistical analyses, SAS 9.4 software will be used.

References

1. Localio AR, Berlin JA, Ten Have TR, Kimmel SE. Adjustments for center in multicenter studies: an overview. Ann Intern Med. 2001;135(2):112-23.
2. Shao J, Zhong B. Last observation carry-forward and last observation analysis. Stat Med. 2003;22(15):2429-41.
3. Sterne JA, White IR, Carlin JB, Spratt M, Royston P, Kenward MG, et al. Multiple imputation for missing data in epidemiological and clinical research: potential and pitfalls. BMJ. 2009;338:b2393.
