# Supplementary material for: Acupuncture for menstruation-related migraine prophylaxis: A multicenter randomized controlled trial
Source: Front Neurosci. 2022 Aug 26;16:992577. doi: 10.3389/fnins.2022.992577 (PMC9459087; doi:10.3389/fnins.2022.992577)
Supplement: Supplementary file 2 [file Data_Sheet_2.PDF]

Supplemental Material 2

Figure 1. Location of traditional acupoints in the acupuncture group and non-effective acupoints in the medication group

Table 1. Fifteen acupoints without effects on headache or menstruation

Table 2. Three subgroups of the non-effective acupoints in the medication group

Appendix 1. Multiple Imputation

Table 3. Missing Data Patterns

Appendix 2. Sensitivity analysis using a multiple imputation method with missing data for the primary outcome

Table 4. Sensitivity analysis assuming data missing at random with regression-based multiple imputation model for the primary outcome

Table 5. Demographics and baseline characteristics of 157 patients included in the Per-Protocol analysis

Table 6. Primary and secondary outcomes (Per-Protocol population)

Table 7. Reasons for excluding 192 patients after enrollment and before randomization

Appendix 3. Sensitivity analysis using the individual menstrual cycle length as a control variable for the primary and secondary outcomes

Table 8. Descriptive statistics on the numbers of menstrual cycle and menstruation

Table 9. Between-group comparison of change from baseline in menstrual cycle over cycles 1-3 (treatment phase) and cycles 4-6 (follow-up phase)

Table 10. Within-group comparison of menstrual cycle from baseline at cycles 1-3 (treatment phase) and cycles 4-6 (follow-up phase)

Table 11. Sensitivity analysis controlling for (individual) menstrual cycle length with mixed-effect model for the primary and secondary outcomes

Table 12. Summary of major protocol deviations

Figure 1. Location of traditional acupoints in the acupuncture group and non-effective acupoints in the medication group

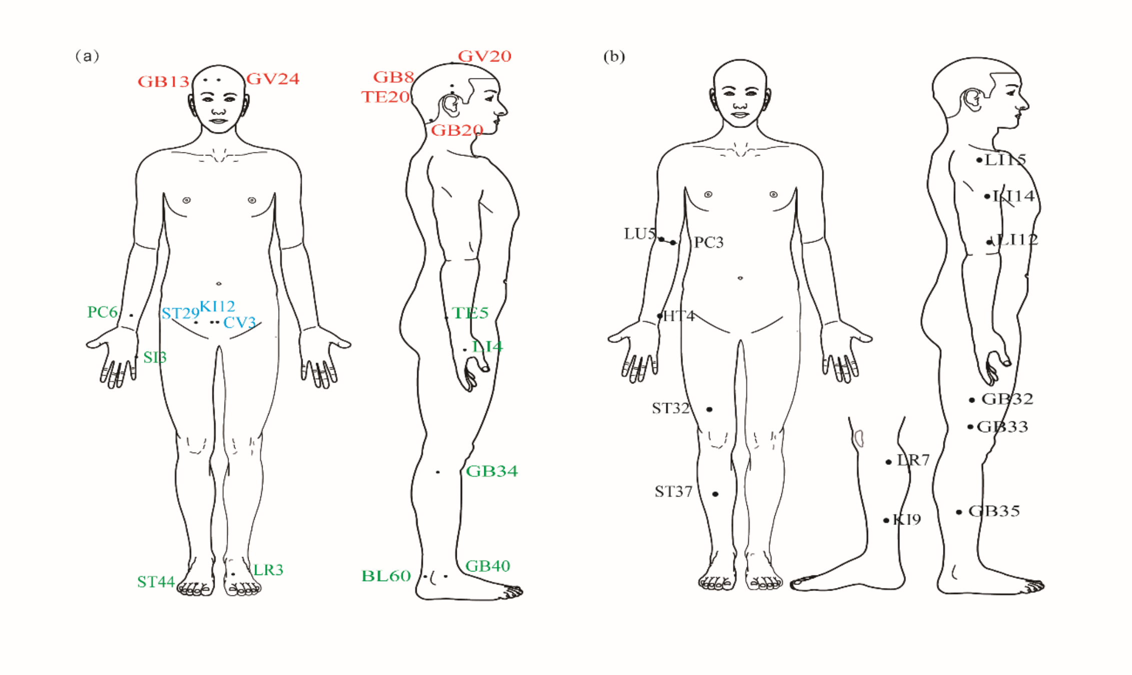

(a) Location of traditional acupoints. Standard points: GV20, GV24, GB13, GB8, TE20, GB20 (Red). Additional points for the syndrome differentiation of meridians: TE5, GB34, LI4, ST44, BL60, SI3, LR3, GB40, PC6 (Green). Premenstrual conditioning points: KI12, CV3, ST29 (Blue). (b) Location of non-effective acupoints.

Table 1. Fifteen acupoints without effects on headache or menstruation

Arm	LU5	LI12	LI14	LI15	HT4	PC2	PC3	TE8
Leg	ST32	ST37	KI9	GB32	GB33	GB35	LR7	

Table 2. Three subgroups of the non-effective acupoints in the medication group

Subgroup	Code	Arm		Leg		
1	B	LU5	HT4	KI9	GB33	GB35
2	C	LI15	PC3	GB35	LR7	ST32
3	D	LI12	LI14	GB32	ST37	KI9

Appendix 1. Multiple Imputation

For the average number of migraine days per perimenstrual period over cycles 1-3 (primary outcome), we had 13 cases with missing data (see eTable 5). The 'Missing Data Patterns' table lists distinct missing data patterns with corresponding frequencies in each group. Here, a 'Pattern' means a unique missing pattern from the data set; values of '√' and '×' indicate that the variable is observed or missing, respectively, in the corresponding pattern. The table confirms a monotone missing pattern for these four time points. We assumed the data were missing at random, and regression-based multiple imputation with baseline the average number of migraine days per perimenstrual period, age and duration of migraine as covariates was used (SAS PROC MI).

Table 3: Missing data patterns

Pattern	Baseline	Cycle 1	Cycle 2	Cycle 3	Acupuncture group (n = 86) n (%)	Medication group (n = 84) n (%)	Total (n = 170) n (%)
1	√	√	√	√	80 (93.02)	77 (91.67)	157 (92.35)
2	√	√	×	×	6 (6.98)	7 (8.33)	13 (7.65)

Note: √ = complete data. × = incomplete data.

Appendix 2. Sensitivity analysis using a multiple imputation method with missing data for the primary outcome

The sensitivity analysis with a regression-based multiple imputation model, assessed whether the change from baseline in the average number of migraine days per perimenstrual period over cycles 1-3 was robust to departure from imputed missing data using the last observation carried forward (LOCF) method. SAS PROC MI with the missing at random statement was used. The results were robust to departure from imputed missing data using the LOCF method (Table 2 and eTable 6).

Table 4. Sensitivity analysis assuming data missing at random with regression-based multiple imputation model for the primary outcome (Intention-to-treat population)[a]

Outcome	Acupuncture group (n = 86)	Medication group (n = 84)	Between-group difference	
			Value (95% CI)	*P* value
Change from baseline in average number of migraine days per perimenstrual period[b], cycles 1-3, mean (95% CI)	1.01 (0.90 to 1.13)	0.66 (0.54 to 0.78)	0.35 (0.19 to 0.52)	<.0001

[a]Mixed-effect mode was used on the intention-to-treat population with regression-based multiple imputation under the missing at random assumption for 13 participants (6 in the acupuncture group and 7 in the medication group); [b]Baseline is the cycle average over the 3-cycle screening phase prior to receiving treatment. CI: confidence interval.

Table 5. Demographics and baseline characteristics of 157 patients included in the Per-Protocol analysis

Characteristics	Acupuncture group n = 80	Medication group n = 77	Total n = 157
Mean (SD) age, y	36.38 (6.52)	35.39 (36.94)	35.89 (6.67)
Mean (SD) duration of migraine diagnosis at baseline, y	7.96 (6.65)	8.51 (5.60)	8.24 (6.14)
Family history, n (%)	24 (30.00)	25 (32.47)	49 (31.21)
Accompanying symptoms:			
Nausea or vomiting, n (%)	77 (96.25)	74 (96.10)	162 (96.18)
Photophobia or phonophobia, n (%)	51 (63.75)	47 (61.04)	98 (62.42)
Others, n (%)	19 (23.75)	15 (19.48)	34 (21.66)
Dysmenorrhea, n (%)	26 (32.50)	19 (24.68)	45 (28.66)
Mean (SD) menstrual cycle, day	29.20 (2.28)	28.74 (2.21)	28.97 (2.25)
Mean (SD) menstruation, day	5.54 (1.15)	5.61 (1.11)	5.57 (1.13)
Mean (SD) number of migraine days during perimenstrual period	1.78 (0.68)	1.65 (0.53)	1.72 (0.61)
Mean (SD) number of migraine days during outside perimenstrual period	2.09 (1.47)	1.77 (0.99)	1.94 (1.26)
Mean (SD) migraine hours during perimenstrual period	20.63 (11.93)	20.16 (11.30)	20.40 (11.59)
Mean (SD) migraine hours during outside perimenstrual period	14.68 (9.00)	14.20 (8.08)	14.45 (8.54)
Mean (SD) pain VAS during perimenstrual period	7.24 (1.46)	7.24 (1.45)	7.24 (1.45)
Mean (SD) pain VAS during outside perimenstrual period	6.14 (1.39)	6.00 (1.48)	6.07 (1.43)
Use of acute pain medication, n (%)	57 (71.25)	50 (64.94)	107 (68.15)

SD: standard deviation; VAS: visual analogue scale.

Table 6. Primary and secondary outcomes (Per-Protocol population)

Outcome	Acupuncture group (n = 80)	Medication group (n = 77)	Between-group difference	
			Value (95% CI)	*P* value
**Primary Outcome**				
Average number of migraine days per perimenstrual period, cycles 1-3, mean (95% CI)	0.77 (0.65 to 0.89)	1.01 (0.90 to 1.11)	0.24 (0.08 to 0.40)	0.0033
Change from baseline in average number of migraine days per perimenstrual period[a], cycles 1-3, mean (95% CI)	1.01 (0.89 to 1.13)	0.64 (0.53 to 0.75)	0.37 (0.21 to 0.53)	<.0001
**Secondary Outcomes**				
Change from baseline in average number of migraine days per perimenstrual period, cycles 4-6, mean (95% CI)	0.91 (0.78 to 1.03)	0.46 (0.34 to 0.59)	0.45 (0.27 to 0.62)	<.0001
Change from baseline in average number of migraine days per outside perimenstrual period, mean (95% CI)				
Cycles 1-3	1.16 (0.97 to 1.36)	0.52 (0.41 to 0.63)	0.64 (0.42 to 0.86)	<.0001
Cycles 4-6	1.03 (0.81 to 1.24)	0.32 (0.21 to 0.43)	0.71 (0.47 to 0.95)	<.0001
Change from baseline in mean migraine hours during perimenstrual period[b], mean (95% CI)				
Cycles 1-3	8.19 (6.76 to 9.62)	5.33 (4.09 to 6.58)	2.86 (0.99 to 4.73)	.0033
Cycles 4-6	6.07 (4.73 to 7.41)	3.32 (2.30 to 4.34)	2.75 (1.09 to 4.41)	.0014
Change from baseline in mean migraine hours during outside perimenstrual period, mean (95% CI)				
Cycles 1-3	4.90 (3.87 to 5.94)	2.78 (2.15 to 3.41)	2.12 (0.92 to 3.32)	.0007
Cycles 4-6	3.78 (2.89 to 4.67)	1.62 (0.92 to 2.23)	2.16 (1.04 to 3.29)	.0002

Change from baseline in mean pain VAS during perimenstrual period, mean (95% CI)				
Cycles 1-3	2.71 (2.34 to 3.08)	2.23 (1.93 to 2.54)	0.48 (0.00 to 0.95)	.0504
Cycles 4-6	2.57 (2.24 to 2.90)	1.35 (1.14 to 1.57)	1.22 (0.82 to 1.61)	<.0001
Change from baseline in mean pain VAS during outside perimenstrual period, mean (95% CI)				
Cycles 1-3	2.33 (1.92 to 2.74)	1.93 (1.65 to 2.21)	0.40 (-0.95to 0.89)	.1140
Cycles 4-6	1.56 (1.31 to 1.81)	1.00 (0.75 to 1.24)	0.56 (0.22 to 0.90)	.0016
50% migraine responder rate, participants, n (%)				
Cycles 1-3	54 (67.5%)	36 (46.8%)	20.7 (5.60 to 35.90)	.0086
Cycles 4-6	50 (62.5%)	32 (41.6%)	20.9 (5.65 to 36.23)	.0086
Use of acute pain medication, participants, n (%)				
Cycles 1-3	21 (26.2%)	30 (39.0%)	12.8 (-27.26 to 1.84)	.0891
Cycles 4-6	37 (46.2%)	42 (54.5%)	8.3 (-23.89 to 7.29)	.2987

[a]Baseline is the cycle average over the 3-cycle screening phase prior to receiving treatment; [b]Baseline is the daily average over the 3-cycle screening phase prior to receiving treatment; CI: confidence interval.

Table 7. Reasons for excluding 192 patients after enrollment and before randomization

Reasons for exclusion	Numbers
**Not meeting inclusion criteria**	
Diagnosed as MRM according to ICHD II	47
With regular menstrual cycle	35
Could predict within three days both the onset of menstruation and perimenstrual migraine attacks	16
Has repeated migraine attacks, with the frequency of non-menstrual migraine being more than once a month	13
Provided written informed consent	12
**Meeting exclusion criteria**	
With chronic migraine, tension-type headache, cluster headache and other primary headaches	0
Secondary headache and other neurological diseases	0
Has relatively severe systemic diseases (cardiovascular disease, acute infectious disease, hematopathy, endocrinopathy and allergy)	6
Headache caused by otorhinolaryngological diseases or intracranial pathological changes	0
Oral contraceptives, pregnancy, or lactation period	0
Use of prophylactic migraine medication in the last three months	11
Involved in other clinical trials	0
**Other exclusion reasons**	
Lacked interest in participation	52

Appendix 3. Sensitivity analysis using the individual menstrual cycle length as a control variable for the primary and secondary outcomes

There are between and within-woman differences in menstrual cycle lengths which inherently confound the outcomes. Sensitivity analyses were used to evaluate the potential confounder as follows: Firstly, we found that the menstrual cycle of acupuncture group and medication group had no significant difference at baseline (cycles -3-0, p = 0.1415), treatment (cycles 1-3, p = 0.1609), and follow-up (cycles 4-6, p = 0.1864) phases (**eTable 8**). Secondly, between-group comparisons revealed that there was no significant difference between acupuncture group and medication group in change from baseline in menstrual cycle over cycles 1-3 (treatment phase) and cycles 4-6 (follow-up phase) (cycles 1-3, p = 0.9061; cycles 4-6, p = 0.8616) (**eTable 9**). Thirdly, separate within-group analysis revealed no significant change in menstrual cycle from baseline at cycles 1-3 (acupuncture group, p = 0.7680; medication group, p = 0.8849) and cycles 4-6 (acupuncture group, p = 0.7869; medication group, p = 1.0000) in both acupuncture and medication group (**eTable 10**). After above three steps *t* test analyses, menstrual cycle length as a control variable was added into a mixed-effect model (**eTable 11**) to evaluate whether the changes from baseline in average number of migraine days per perimenstrual period/ outside perimenstrual period, mean migraine hours during/outside perimenstrual period, and mean pain VAS during/outside perimenstrual period over cycles 1-3 and 4-6 were robust to departure from imputed without menstrual cycle length as a control variable. The results were robust to departure from imputed without menstrual cycle length as a control variable (Table 2 and eTable 6).

Table 8. Descriptive statistics on the numbers of menstrual cycle and menstruation (Intention-to-treat population) [a]

Outcome	Acupuncture group (n = 86)	Medication group (n = 84)	Total (n = 170)	*P* value[a]
**Mean (SD) menstrual cycle, day**[b]				
Baseline (three menstrual cycles)	29.24 (2.32)	28.74 (2.15)	28.99 (2.24)	.1415
Treatment phase	29.21 (2.30)	28.72 (2.21)	28.97 (2.26)	.1609
Follow-up phase	29.21 (2.22)	28.74 (2.41)	28.98 (2.32)	.1864
**Mean (SD) menstruation, day**[b]				
Baseline (three menstrual cycles)	5.53 (1.15)	5.49 (1.19)	5.51 (1.16)	.7939
Treatment phase	5.48 (1.00)	5.27 (1.01)	5.37 (1.00)	.1646
Follow-up phase	5.45 (1.10)	5.23 (1.16)	5.34 (1.13)	.1910

[a]All tests were 2-sided. *P* was calculated from *t* test. *P* value of less than 0.05 was considered significant; [b]Average number of menstrual cycle/menstruation (days) over three cycles during baseline/treatment/follow-up phase; SD: standard deviation.

Table 9. Between-group comparison of change from baseline in menstrual cycle over cycles 1-3 (treatment phase) and cycles 4-6 (follow-up phase)

Variable	Acupuncture group (n = 86)	Medication group (n = 84)	Value (95%CI)	Between group *P* value[a]
**Change from baseline in menstrual cycle, cycles 1-3, mean (SD)[b]**	0.03 (1.09)	0.02 (1.00)	0.01 (-0.30 to 0.34)	.9061
**Change from baseline in menstrual cycle, cycles 4-6, mean (SD)[b]**	0.03 (1.19)	0.00 (1.41)	0.03 (-0.36 to 0.43)	.8616

[a]All tests were 2-sided. *P* was calculated from *t* test. *P* value of less than 0.05 was considered significant; [b]Baseline is the cycle average over the 3-cycle screening phase prior to receiving treatment; SD: standard deviation; CI: confidence interval.

Table 10. Within-group comparison of menstrual cycle from baseline at cycles 1-3 (treatment phase) and cycles 4-6 (follow-up phase)

Variable	Group	Baseline phase Cycles -3-0	Treatment phase Cycles 1-3	Within group *P* value[a]	Baseline phase Cycles -3-0	Follow-up phase Cycles 4-6	Within group *P* value[a]
**Mean (SD) menstrual cycle, day[b]**	Acupuncture group (n = 86)	29.24 (2.32)	29.21 (2.30)	0.7680	29.24 (2.32)	29.21 (2.22)	.7869
	Medication group (n = 84)	28.74 (2.15)	28.72 (2.21)	0.8849	28.74 (2.15)	28.74 (2.41)	1.0000

[a]All tests were 2-sided. *P* was calculated from paired *t* test. *P* value of less than 0.05 was considered significant; [b]Average number of menstrual cycle (days) over three cycles during baseline/treatment/follow-up phase; SD: standard deviation.

Table 11. Sensitivity analysis controlling for (individual) menstrual cycle length with mixed-effect model for the primary and secondary outcomes (Intention-to-treat population)[a]

Outcome	Acupuncture group (n = 86)	Medication group (n = 84)	Between-group difference	
			Value (95% CI)	*P* value[b]
**Primary Outcome**				
Average number of migraine days per perimenstrual period[c], cycles 1-3, mean (95% CI)	0.89 (0.74 to 1.05)	1.06 (0.95 to 1.17)	0.17 (-0.24 to 0.36)	.0862
Change from baseline in average number of migraine days per perimenstrual period[d], cycles 1-3, mean (95% CI)[e]	0.94 (0.82 to 1.07)	0.61 (0.50 to 0.71)	0.33 (0.17 to 0.49)	<.0001
**Secondary Outcomes**				
Change from baseline in average number of migraine days per perimenstrual period, cycles 4-6, mean (95% CI)[e]	0.84 (0.72 to 0.97)	0.44 (0.32 to 0.56)	0.41(0.24 to 0.58)	<.0001
Change from baseline in average number of migraine days per outside perimenstrual period, mean (95% CI)[e]				
Cycles 1-3	1.08 (0.89 to 1.27)	0.49 (0.39 to 0.60)	0.57 (0.36 to 0.79)	<.0001
Cycles 4-6	0.95 (0.75 to 1.16)	0.31 (0.21 to 0.41)	0.65 (0.42 to 0.88)	<.0001
Change from baseline in mean migraine hours during perimenstrual period[f], mean (95% CI)[e]				
Cycles 1-3	7.61 (6.22 to 9.02)	5.01 (3.83 to 6.20)	2.42 (0.62 to 4.22)	.0090
Cycles 4-6	5.65 (4.36 to 6.93)	3.09 (2.14 to 4.05)	2.58 (0.99 to 4.17)	.0020
Change from baseline in mean migraine hours during outside perimenstrual period[f], mean (95% CI)[e]				

Cycles 1-3	4.56 (3.56 to 5.66)	2.59 (1.99 to 3.19)	1.86 (0.72 to 3.00)	.0010
Cycles 4-6	3.52 (2.66 to 4.37)	1.51 (0.86 to 2.15)	2.01 (0.95 to 3.07)	<.0001
Change from baseline in mean pain VAS during perimenstrual period, mean (95% CI)[e]				
Cycles 1-3	2.52 (2.15 to 2.90)	2.14 (1.84 to 2.44)	0.37 (-0.11 to 0.84)	.1290
Cycles 4-6	2.39 (2.05 to 2.73)	1.30 (1.09 to 1.52)	1.08 (0.69 to 1.48)	<.0001
Change from baseline in mean pain VAS during outside perimenstrual period, mean (95% CI)[e]				
Cycles 1-3	2.17 (1.76 to 2.57)	1.81 (1.53 to 2.09)	0.35 (-0.14 to 0.83)	.1590
Cycles 4-6	1.45 (1.20 to 1.69)	0.95 (0.71 to 1.18)	0.50 (0.16 to 0.83)	.0040
50% migraine responder rate, participants, n (%)[g]				
Cycles 1-3	54 (62.8%)	36 (42.9%)	19.9 (5.22 to 34.64)	.0092
Cycles 4-6	50 (58.1%)	32 (38.1%)	20.0 (5.33 to 34.76)	.0089
Use of acute pain medication, participants, n (%)[g]				
Cycles 1-3	24 (27.9%)	35 (41.7%)	13.8 (-27.94 to 0.42)	.0595
Cycles 4-6	40 (46.5%)	46 (54.8%)	8.3 (-23.23 to 6.73)	.2821

[a]Average number of migraine days per perimenstrual period/outside perimenstrual period, mean migraine hours during/outside perimenstrual period, and mean pain VAS during/outside perimenstrual period were missing in 6 participants of the acupuncture group and 7 participants of the medication group over cycles 1-3, and also in 6 participants of the acupuncture group and 7 participants of the medication group over cycles 4-6. The missing data of participants who dropped out were replaced using the last observation carried forward method. Number of participants with imputed data: 6 (7.0%) of the acupuncture group, and 7 (8.3%) of the medication group; [b]All tests were 2-sided. *P* value of less than 0.05 was considered significant; [c] Perimenstrual period is from the two days before menstruation through first three days of menstruation; [d]Baseline is the cycle average over the 3-cycle screening phase prior to receiving treatment; [e]Analyzed by fitting a mixed-effect model using baseline value as a covariate, menstrual cycle length as a control variable, treatment as a fixed effect, and center as a random effect; [f]Baseline is the daily average over the 3-cycle screening phase prior to receiving treatment; [g]Analyzed using Chi-square test; CI: confidence interval; VAS: visual analogue scale.

Table 12. Summary of major protocol deviations

Group	Finished trial (Yes/No)	Reasons of dropped out	Category of deviation	Reasons of major PDs as reported by investigators	Treatment sessions
Acupuncture group	No	Time restriction	Study Procedure	Missed visit/assessment	8
Acupuncture group	No	Time restriction	Study Procedure	Missed visit/assessment	8
Acupuncture group	No	Fear of needling	Study Procedure	Missed visit/assessment	8
Acupuncture group	No	Time restriction	Study Procedure	Missed visit/assessment	9
Acupuncture group	No	Time restriction	Study Procedure	Missed visit/assessment	10
Acupuncture group	No	Time restriction	Study Procedure	Missed visit/assessment	11
Medication group	No	Time restriction	Study Procedure	Missed visit/assessment	8
Medication group	No	Time restriction	Study Procedure	Missed visit/assessment	8
Medication group	No	Unsatisfied	Study Procedure	Missed visit/assessment	8
Medication group	No	Residence changed	Study Procedure	Missed visit/assessment	9
Medication group	No	Fear of needling	Study Procedure	Missed visit/assessment	8
Medication group	No	Time restriction	Study Procedure	Participant seen outside window period	11
Medication group	No	Time restriction	Study Procedure	Missed visit/assessment	8

PD: protocol deviations.
